# Comparison of Photocatalytic and Adsorption Properties of ZnS@ZnO, CdS@ZnO, and PbS@ZnO Nanocomposites to Select the Best Material for the Bifunctional Removal of Methylene Blue

**DOI:** 10.1021/acsomega.4c07910

**Published:** 2025-03-04

**Authors:** Ümit Bayram, Çiğdem Özer, Erkan Yilmaz

**Affiliations:** † Central Research Facility (AGU-CRF), Abdullah Gül University, 38080 Kayseri, Turkey; ‡ Erciyes University Nanotechnology Application and Research Center (ERNAM), 38039 Kayseri, Turkey; § Faculty of Pharmacy, Erciyes University, 38039 Kayseri, Turkey; ∥ Technology Research & Application Center (TAUM), Erciyes University, 38039 Kayseri, Turkey; ⊥ Erciyes Teknopark ChemicaMed Chemical Inc., Erciyes University Technology Development Zone, 38039 Kayseri, Turkey

## Abstract

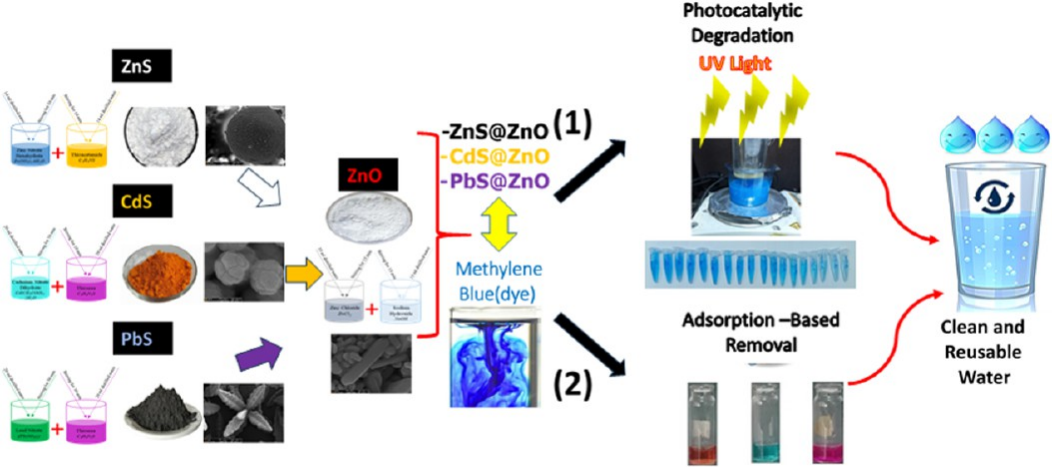

In this study, photocatalytic- and adsorption-based removal processes were conducted, which are frequently preferred in wastewater treatment due to their ease of control and high removal efficiency. An innovative method aimed at wastewater treatment was developed by combining the advantages of these two distinct approaches within the same material. The study synthesized ZnO, ZnS, CdS, PbS, and their composite structures (ZnS@ZnO, CdS@ZnO, and PbS@ZnO) using a hydrothermal synthesis method. Characterization of the samples was performed through field emission-scanning electron microscopy (FE-SEM), FE-SEM-energy dispersive X-ray (FE-SEM-EDX), X-ray diffraction (XRD), Raman spectroscopy, and Fourier-transform infrared spectroscopy (FTIR) measurement. Additionally, the optical properties of all samples (absorption spectra and band gap) were investigated by using absorbance measurements obtained from ultraviolet (UV)–visible absorption spectroscopy. Although ZnO nanoparticles are among the materials with high photocatalytic properties (exhibiting a photodegradation efficiency of 95.8% in a short duration of 90 min), their adsorption properties are low. Therefore, with the aim of enhancing both the low adsorption values and the photocatalytic properties of pure metal sulfides (ZnS, CdS, PbS), nanocomposites ZnS@ZnO, CdS@ZnO, and PbS@ZnO with different morphologies were synthesized, and their photocatalytic and adsorption-based removal performances on methylene blue (MB) dye were investigated. FE-SEM images indicated that ZnS nanoparticles exhibit a spherical morphology, CdS nanoparticles have a flower-like morphology, and PbS nanoparticles display a dendritic-like structure. The results obtained from experimental studies demonstrated that the highest efficiency in both photocatalytic- and adsorption-based removal was achieved with the ZnS@ZnO nanocomposite. The degradation rates of MB were found to be 95.3, 90.5, and 89.4% for the heterojunction composites ZnS@ZnO, CdS@ZnO, and PbS@ZnO, respectively, over a time range of 0–480 min. The optimal amount of photocatalyst that could effectively degrade MB was determined to be 100 mg, and the reusability studies revealed that the ability of the ZnS@ZnO semiconductor heterojunction photocatalyst to decompose MB into simpler molecules was limited after the fourth cycle. The adsorption-based removal rates were 96.0, 30.5, and 19.4% for the heterojunction composites ZnS@ZnO, CdS@ZnO, and PbS@ZnO, respectively. Finally, parameters influencing the adsorption-based removal of MB, such as pH, mass, and contact time, were examined, indicating that the adsorption capacity of ZnS@ZnO remained unchanged after reaching a value of 40 mg·g^–1^.

## 1 Introduction

In today’s conditions, there is an important need for the development of water treatment technologies to ensure quality water, remove chemical and biological pollutants from industrial wastewater, and protect the environment from pollution. The release of untreated waste into water resources leads to the destruction of aquatic life and the disruption of ecological balance. It also results in various diseases and health problems in humans. For example, the dye and textile industries are among the largest contributors to water pollution. From this perspective, advanced wastewater treatment processes involving semiconductor nanoparticles are regarded as green technologies or sustainable mechanisms due to their utilization of renewable (solar) energy sources. Moreover, the removal of pollutants through photocatalytic methods using semiconductors has garnered significant interest because of its potential practical applications. Since the study conducted by Fujishima and Honda^1^ in 1972 using TiO_2_, semiconductors have been widely employed in photocatalytic research. Approximately 45% of the energy from sunlight falls within the energy range of 1.7–2.5 eV, which is applicable to many other semiconductor materials, including metal sulfides, oxides, oxysulfides, and oxynitrides.^2^ Additionally, metal oxides and sulfides such as TiO_2_, ZnS, ZnO, Fe_2_O_3_, PbS, WS_2_, ZrO_2_, CdS, WO_3_, V_2_O_5_, and CeO_2_ are of great interest due to their potential practical applications in the photocatalytic removal of pollutants using semiconductors.^3^

In this study, ZnO, ZnS, CdS, PbS, and their composite structures (ZnS@ZnO, CdS@ZnO, and PbS@ZnO) were synthesized by using the hydrothermal method to investigate their photocatalytic activities and adsorption-based dye removal capacities. Among these materials, ZnS exhibits superior catalytic properties compared to TiO_2_ and ZnO due to the significant negative reduction potential of the excited electrons, and electron–hole pairs are generated rapidly after photoexcitation.^4^ ZnS, which is a nontoxic semiconductor, is used as a photocatalyst for the removal of organic pollutants by the photodegradation process and is known to show good photostability under UV irradiation.^5,6^ Pure ZnS has a wide band gap (3.68 eV)^7^ and therefore can only be activated by excitation with light photons in the UV region. To overcome this limitation and increase the efficiency of ZnS under solar radiation, it is necessary to modify it with nanomaterials.

Cadmium sulfide (CdS) is a semiconductor that can be activated by visible radiation due to its very narrow direct band gap energy (2.4 eV) and exhibits more visible light absorption than TiO_2_.^8^ Also, the conduction band position of CdS is negative enough to meet the thermodynamic conditions for many redox reactions.^9−11^ CdS, which is still a very actively used nanomaterial, has found applications in the manufacture of thin-film transistors, flat panel displays, solar cells, photocatalysts, and light-emitting diodes.^12−15^ In addition, CdS is one of the third-generation semiconductors, and several studies report the development of self-powered integrated gas sensors based on it.^16,17^ The success of these sensors is attributed to the simultaneous presence of piezoelectric and gas-sensing capabilities.^18^

Lead sulfide (PbS) is one of the semiconductor metal sulfides that show excellent electronic and optical properties in the cubic rock salt structure.^19^ The band gap of PbS, which has a bulk band gap of 0.41 eV at room temperature, can be increased up to approximately 2.0 eV by forming different modifications and nanoclusters.^20^ Besides being known as a good material in the field of photocatalysis, PbS continues to be used and developed due to its applications in different fields: Solar absorption,^21^ IR region detectors,^22,23^ advanced optoelectronic devices,^24−26^ and photonic materials.^27^ Additionally, PbS is used to impart sensing behavior to wireless self-powered NO_2_ gas sensors.^28^

ZnO, an n-type semiconductor oxide, has almost the same energy band gap as TiO_2_ (3.2 eV). It is recommended as an alternative photocatalyst to TiO_2_ since it has higher absorption efficiency in a large part of the solar radiation spectrum compared to TiO_2_.^29,30^ In addition, ZnO not only has antibacterial and very good photocatalytic effect but also has excellent optical, mechanical, and electrical properties.^31,32^ ZnO is a versatile semiconductor material that can be easily synthesized in various nanostructures, including those with different dimensions. Thus, ZnO morphology can be used to control the fundamental physical and chemical properties of the structures under investigation.^33^ Due to its existing properties, it is possible to synthesize hybrid sulfide nanostructures by using ZnO to increase the photocatalytic activity in metal sulfides such as ZnS, PbS, and CdS.

In this article, ZnO, ZnS, CdS, and PbS semiconductor materials were synthesized using the hydrothermal method due to their low cost and simplicity. After completing the photodegradation experiments of each of the metal sulfides alone, it was aimed to increase the photocatalytic activities by modifying them with ZnO. All obtained semiconductor materials were analyzed by taking field emission-scanning electron microscopy (FE-SEM), FE-SEM-energy dispersive X-ray (FE-SEM-EDX), X-ray diffraction (XRD), Raman Spectra, and Fourier-transform infrared spectroscopy (FTIR) measurements. An evaluation of the results demonstrating the dual-functional behavior of the heterojunction nanocomposites (ZnS@ZnO, CdS@ZnO, and PbS@ZnO) was conducted. Among these, ZnS@ZnO exhibited the highest photocatalytic activity; therefore, studies were primarily focused on its photocatalytic mechanism, optimal material quantity, and reusability. Experimentally, the adsorption-based removal rates of the heterojunction composites were determined, and subsequently, parameters influencing the adsorption-based removal of the methylene blue (MB), such as pH, mass, and contact time, were optimized to establish the adsorption capacity of ZnS@ZnO. All obtained results were also compared with existing models and relevant studies in the literature.

## 2 Materials and Methods

### 2.1 Materials and Equipment

Zinc nitrate hexahydrate ((Zn(NO_3_)_2_)·6H_2_O), thioacetamide (C_2_H_5_NS), and zinc chloride (ZnCl_2_) were purchased from the Merck company (Merck, Darmstadt, Germany). Sodium hydroxide (NaOH), cadmium acetate dihydrate (Cd(CH_3_COO)_2_·2H_2_O), lead nitrate (Pb(NO_3_)_2_), and thiourea (CH_4_N_2_S) were obtained from Sigma-Aldrich (St. Louis, MO). Deionized water, with resistivity of 18.2 MΩ·cm, was obtained from the Milli-Q system (Millipore). In addition, no further purification methods were used for these materials, and then they were added to the solutions. Determination of methylene blue that is used as a target analyte was performed with an ultraviolet–visible spectrophotometer from PerkinElmer (Lambda 25; Norwalk, CT). Methylene blue stock solution (1.0 × 10^–3^ M) was prepared in analytical grade ethanol. A pH meter and a shaker mixer were used for adsorption-based removal studies. A centrifuge device was used for the isolation and separation of the nanocomposite.

### 2.2 Synthesis with Hydrothermal Method and Preparation of Semiconductor Materials

In this study, all photocatalysts were obtained using the hydrothermal method. Toward this objective, the hydrothermal synthesis unit used (hydrothermal autoclave reactor) consists of six main components: A Teflon liner and five parts made of stainless steel (kettle body, rod, chassis, gland, and lid). It is constructed from stainless steel to operate under high pressure and temperature, with the outer shell made of Teflon and the inner lining fabricated from high-purity poly(tetrafluoroethylene) (PTFE) (see the Graphical Abstract, Autoclave 75 mL). The sealing mechanism for the circular chassis and gland is reinforced with a manual screw. The applicable operating temperature and pressure are a maximum of 250 °C and 3.0 MPa, respectively.

In this first part, zinc nitrate hexahydrate solution in 15 mL of deionized water and thioacetamide solution in 15 mL of deionized water were allowed to dissolve in separate beakers for 30 min in a 1:1 ratio. Then, the thioacetamide solution was added just drop by drop into the zinc nitrate solution and stirred for 60 min until it was completely dissolved to produce the transparent solution. After that, the resulting solution was poured into an autoclave with a capacity of 75 mL, placed in the hydrothermal synthesis unit, and kept in an oven at 100 °C for 24 h to obtain ZnS semiconductor photocatalysts. After the heating period, the synthesis unit was allowed to cool down to room temperature, and after centrifugation at 4000 rpm for 10 min, the white powder was collected. Finally, the resulting powder was washed several times with deionized water and ethanol, and after drying for 24 h in an oven at 50 °C, ZnS powder samples were obtained.

ZnO nanoparticles were used as additives to increase the photocatalytic effect of sulfur-based nanostructured particles. The summary of ZnO synthesis steps is as follows; First, 2 g of zinc chloride (ZnCl_2_) solution in 25 mL of deionized water and 5 g of sodium hydroxide (NaOH) solution in 25 mL of deionized water were dissolved in separate beakers for 30 min in a mixer. Afterward, the NaOH solution was added dropwise into the ZnCl_2_ solution and mixed for 45 min until a homogeneous solution was obtained. This final solution was poured into the autoclave, put into the synthesis unit, and kept in an oven at 180 °C for 12 h. In the last step, ZnO powder samples were obtained by washing with distilled water and ethanol and drying in an oven at 60 °C for 6 h.

Afterward applying similar experimental steps in obtaining ZnS nanoparticles, the solution was prepared by adding 100 mg of ZnO nanoparticles, and ZnS@ZnO nanostructured particles were obtained by using the hydrothermal synthesis method. ZnO and ZnS samples were prepared in separate beakers, and the ZnS solution was added drop by drop into the beaker containing 100 mg of ZnO in 10 mL of deionized water and mixed for 1 h. After obtaining a homogeneous mixture, it was poured into the autoclave, placed in the synthesis unit, and kept in an oven at 100 °C for 24 h. Finally, the ZnS@ZnO-based powder sample was obtained by washing several times with distilled water and ethanol, centrifuging again, and drying in an oven at 60 °C for 24 h. The hydrothermal synthesis method was applied under similar experimental conditions for PbS, PbS@ZnO, CdS, and CdS@ZnO samples, and the experimental procedures and chemicals are shown in Table 1 and Supporting Information Figure S1.

**Table 1:** Experimental Conditions and Chemicals for Semiconductor Nanoparticles Obtained by the Hydrothermal Synthesis Method

				hydrothermal synthesis		drying process	
sample	chemicals			time (h)	temperature (°C)	time (h)	temperature (°C)
ZnS	zinc nitrate hexahydrate Zn(NO_3_)_2_·6H_2_O		thioacetamide C_2_H_5_NS	24	100	24	50
PbS	lead nitrate (Pb(NO_3_)_2_)		thiourea C_4_H_4_N_2_S	16	150	12	60
CdS	cadmium nitrate dihydrate Cd(CH_3_COO)_2_·2H_2_O		thiourea C_4_H_4_N_2_S	12	160	12	80
ZnO	zinc chloride ZnCl_2_		sodium hydroxide NaOH	12	180	6	60
ZnS@ZnO	zinc nitrate hexahydrate	thioacetamide	100 mg ZnO	24	100	24	50
PbS@ZnO	lead nitrate	thiourea	100 mg ZnO	24	150	12	80
CdS@ZnO	cadmium nitrate dihydrate	thiourea	100 mg ZnO	12	160	12	80

### 2.3 Characterization of Powder Samples

A powder X-ray diffractometer (XRD Bruker, Discover 8, Germany), operated at 40 kV and 40 mA and with graphite monochromatized Cu-Kα1 radiation (λ = 0.15406 nm) as the X-ray source, was used to detect the crystallographic patterns of the photocatalysts; the scan range (2θ) was formed from 15 to 80°. To characterize all samples and define each crystal phase, by exposing them to an X-ray beam of known wavelength, a diffraction pattern specific for each crystal phase was produced, and with these patterns, Miller indices (*hkl*) and component amounts were obtained from the Diffrac Suite EVA software equipped with the current ICDD PDF-2 database connected to the XRD device.

The surface morphological features of the samples were examined by field emission scanning electron microscopy (FE-SEM, Zeiss Gemini SEM300, Germany). Before taking FE-SEM images, they were coated with 5 nm thick gold with a desk sputter coater (Quorum, Q150R Plus, Great Britain) to improve the resolution of the images. A confocal Raman spectrometer (WITec Alpha300, Germany) equipped with a 50× Leica microscope objective (NA 0.85) operating under laser excitation with a wavelength of 532 nm and a power of 3.2 mW was used to obtain the Raman spectra of the samples. The integration time was set to 10 s. Aqueous MB solutions of different concentrations were dropped on the substrates, and Raman spectra were taken after the evaporation of the water. To increase the sensitivity of the measurements, spectra were obtained from ten different positions on the substrate. Finally, to analyze the bond characteristics of all synthesized catalysts, FTIR measurements were performed over a range of 2000–580 cm^–1^ using attenuated total reflection (ATR) mode.

### 2.4 Determination of Photocatalytic Activity and Degradation Rates

Photocatalytic degradation behavior of semiconductor materials synthesized against MB under visible light was revealed by a comparative study. 100 mg of each ZnO, ZnS, PbS, and CdS sample was added separately to 150 mL of MB solution (500 mg/500 mL of deionized (DI) water-stock solution) and stirred overnight (approximately 8 h) in the dark to reach adsorption–desorption equilibrium. Visible light irradiation was performed using a 400 W halogen lamp. To cool the lamp, the halogen lamp was enclosed in a glass tube surrounded by a circulating water tube. 1 mL of reaction solutions was pipetted into a quartz cell at regular intervals of 30 min and analyzed by UV–visible spectrometry to examine the change in initial dye concentration in which MB was selected as the probe molecule after centrifugation. The absorbance spectra of the solutions before the start of the irradiation were also obtained. UV–visible spectroscopy measurements for all samples (Lambda 25, PerkinElmer) were performed in the 400–800 nm wavelength range. Spectra and baselines (ultrapure water) were recorded with a 1 nm resolution at 25 °C. The wavelength at the maximum peak for MB is 664 nm, and the reduction in absorption for this wavelength was measured as a function of the photodegradation time. To increase the photocatalytic activity of ZnS, CdS, and PbS semiconductor nanoparticles, 100 mg of ZnO was added to each solution separately (Table 1). The absorbance spectra of the ZnS@ZnO, PbS@ZnO, and CdS@ZnO samples were recorded by repeating the experimental steps performed in the previous stage.

The percentage of degradation ratio values were obtained with the help of the following equation
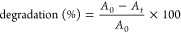
1where *A*_0_ is the initial absorbance of the probe molecule and *A_t_* is the absorbance of the probe molecule after “*t*” min.

Also, the pseudo-first-order chemical kinetics reaction is represented by the following eq 2
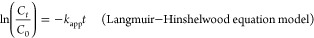
2where *k*_app_ (min^–1^), the apparent reaction rate constant, is calculated from the slope of the graphical regression line of ln(*C_t_*/*C*_0_). In eq 2, *C_t_* and *C*_0_ express the concentration of probing molecules at the adsorption equilibrium concentration at time *t* (min) and at the initial irradiation time, respectively. In addition to these explanations, the time required to reduce the initial concentration value to half (*t*_1/2_-half-life time) and eqs 3–5 can be used to calculate the apparent reaction rate constant.
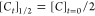
3
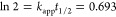
4
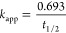
5

### 2.5 Determination of Degradation Rates Based on Adsorption Activities

To evaluate the adsorption performance of MB, adsorption experiments were carried out 3 times under the same conditions, at room temperature, at different pH values ranging from 4 to 10. In the experiment, 25 mg of each material was immersed in 20 μL of MB solution (completed with 10 mL of H_2_O). Here, the initial pH was adjusted with a buffer solution. It was separated from the sample solution after centrifugation by stirring with a shaker mixer for 30 min, and the residual MB amounts were determined by a UV–vis spectrophotometer at λ_max_ = 664 nm.

The percentage of adsorption is given by the following equation
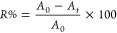
6where *A*_0_ and *A_t_* are the absorption value of the prepared reference solution and the absorption value of the solution at the end of the process, respectively.

The effect of contact time on the adsorption-based removal of MB was evaluated using 50 mg of adsorbent at pH 6. A volume of 1 mL was taken from the sample solutions at different contact times such as 10, 20, 30, and 40 min. The solution is then analyzed by UV–vis to determine the amount of MB remaining.

The adsorption capacity of the material was calculated using the following equation
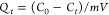
7where *Q_t_* (mg·g ^–1^) is the equilibrium adsorption capacity, *C*_0_ (mg·L^–1^) and *C_t_* (mg·L ^–1^) are the initial and equilibrium concentration of MB, respectively, *V* (L) is the volume of MB solution, and *m* (g) is the mass of the adsorbent.

## 3 Results and Discussion

### 3.1 XRD Results Assessment

**Figure 1:**
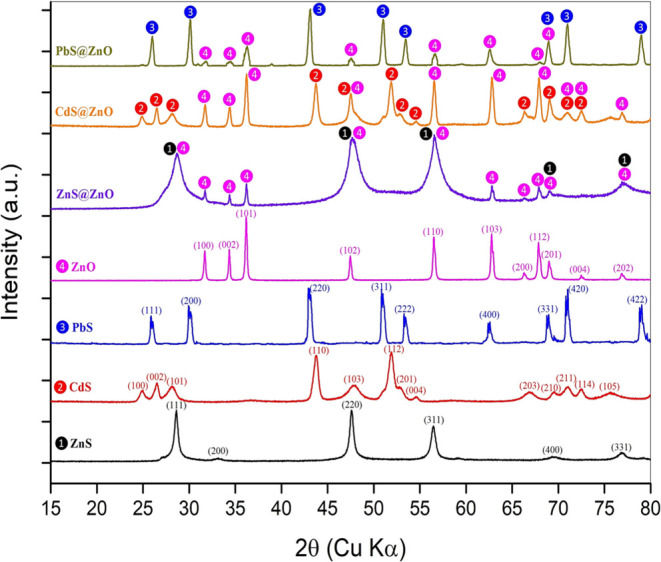
XRD diffractometer pattern of ZnS, CdS, PbS, ZnO, ZnS@ZnO, CdS@ZnO, and PbS@ZnO nanoparticles.

The X-ray analysis method reveals very useful information about the identification of phases, chemical compositions, and crystallographic structure of materials with a crystalline structure.^34,35^Figure 1 shows the powder X-ray diffraction (XRD) patterns for all samples obtained by the hydrothermal synthesis method in this study. The pattern of the raw zinc oxide (ZnO) showed good agreement with the model of hexagonal Zincite (wurtzite phase) as the main crystalline phase according to Joint Committee on Powder Diffraction Standards (JCPDS) Card No. 36–1451,^36−38^ and the 11 peaks at 2θ = 31.77, 34.42, 36.25, 47.54, 56.61, 62.86, 66.38, 67.96, 69.11, 72.56, and 76.96 have corresponded to crystallographic planes (100), (002), (101), (102), (110), (103), (200), (112), (201), (004), and (202), respectively. ZnO has a high degree of crystallinity, which can be seen from the intense and sharp diffraction peaks in the diffraction pattern. It can also be seen from the peak with the highest intensity at 36.25° that the synthesized ZnO nanostructures are oriented along the (101) plane. According to the Debye–Scherrer formula used to calculate the average of the crystal size^39−42^
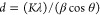
8where β is the peak width (β: full width at half-maximum in radians), λ is the X-ray wavelength in nm (0.15406 nm), θ is the Bragg angle, *d* is a crystallite size, and the *K* value, known as the Scherer constant, varies depending on the crystal shape and takes values between 0.8 and 1.39 (usually 0.94).^43−45^ The Debye–Scherrer formula in eq 8 implies that peak broadening tends to decrease the crystallite size. The most intense peaks in the XRD patterns were cared to calculate the average crystallite size of all samples. In the calculation based on this formula, the average crystallite size of ZnO is 31.08 nm.

Additionally, the XRD patterns obtained for cubic ZnS (zinc blende), hexagonal CdS, and cubic PbS crystallite phases were found to be compatible with standard No. 05–0566 (Sphalerite), No. 41–1049 (Greenockite), and No. 65–9496 (Galena), respectively. The planes 28.56° (111), 47.52° (220), 56.29° (311), and 76.81° (331) observed in ZnS are in good agreement with the cubic phase of ZnS, and from eq 8, the crystal size is 12.78 nm. The crystallite size calculated from diffraction peaks shown in Figure 1 for ZnS@ZnO is not possible due to the presence of mixed peaks of hexagonal ZnO and cubic ZnS. A similar situation applies to CdS@ZnO and PbS@ZnO. From the calculations made based on eq 6, crystal sizes for CdS and PbS were calculated as 9.39 and 21.12 nm, respectively. Detailed information about the XRD patterns obtained for powder samples synthesized in this study is given in Supporting Information Table S1.

**Figure 2:**
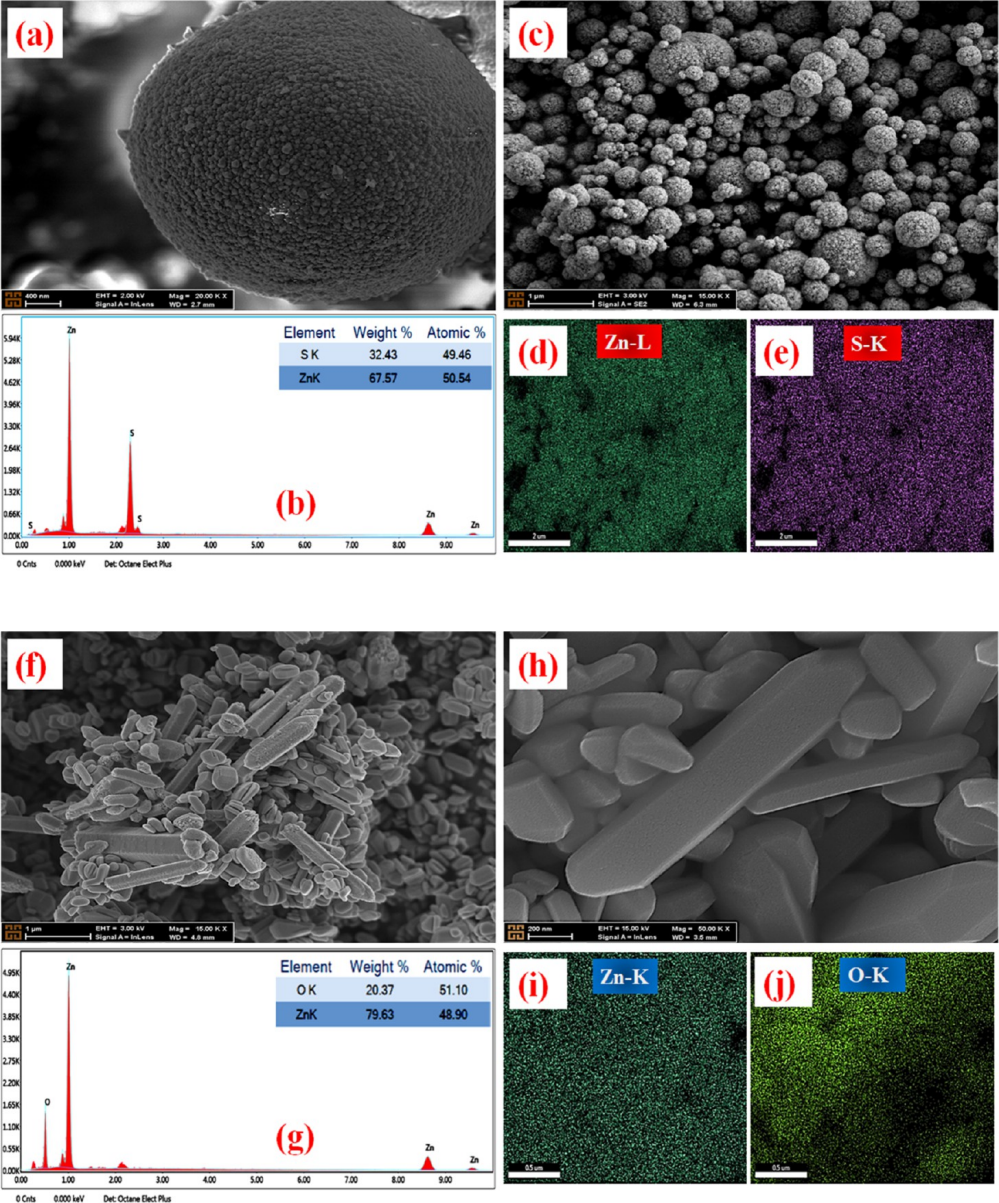
FE-SEM images, EDX elemental analysis, and mapping for morphological characterization with different magnifications for ZnS (a–e) and ZnO (f–j).

FE-SEM was used to analyze the changes in the structure of the materials and the surface morphology of the synthesized powder surfaces. Furthermore, energy-dispersive X-ray spectroscopy (EDX Spectra), an elemental analysis technique, was applied to characterize the elements in the samples, and elemental distribution maps (X-ray maps) were also obtained. Figures 2^4^–5 show FE-SEM images, EDX results, and elemental distribution maps of ZnS, ZnO, CdS, and PbS nanostructures and their composite formations with ZnO.

Figure 2a–e shows the FE-SEM images (a–c), elemental distribution by EDX (b), and X-ray mapping (d–e) results for ZnS. The weight and atomic percentage of the detected elements were also given in the inset table within the EDX graph, and according to the results, the weight percentages of Zn and S were obtained as 32.43 and 67.57, respectively. FE-SEM results of all samples in this study are presented in a similar order. ZnS morphology is submicron spherical (∼3 μm) structures and consists of quasi-spherical particles with sizes varying between 30 and 40 nm. Due to the high amount of thioacetamide (C_2_H_5_NS), it is observed that spherical particles are formed with the release of large amounts of sulfur ions in the solution. Afterward, as a result of the self-assembly of these particles, a structure in the form of a submicrosphere decorated with nanoparticles is formed. In other words, spherical structures have rough surfaces formed by the combination of very small nanoparticles. ZnO (Figure 2f–j) consists mostly of nanorod structures with average sizes of approximately 100–200 nm.

**Figure 3:**
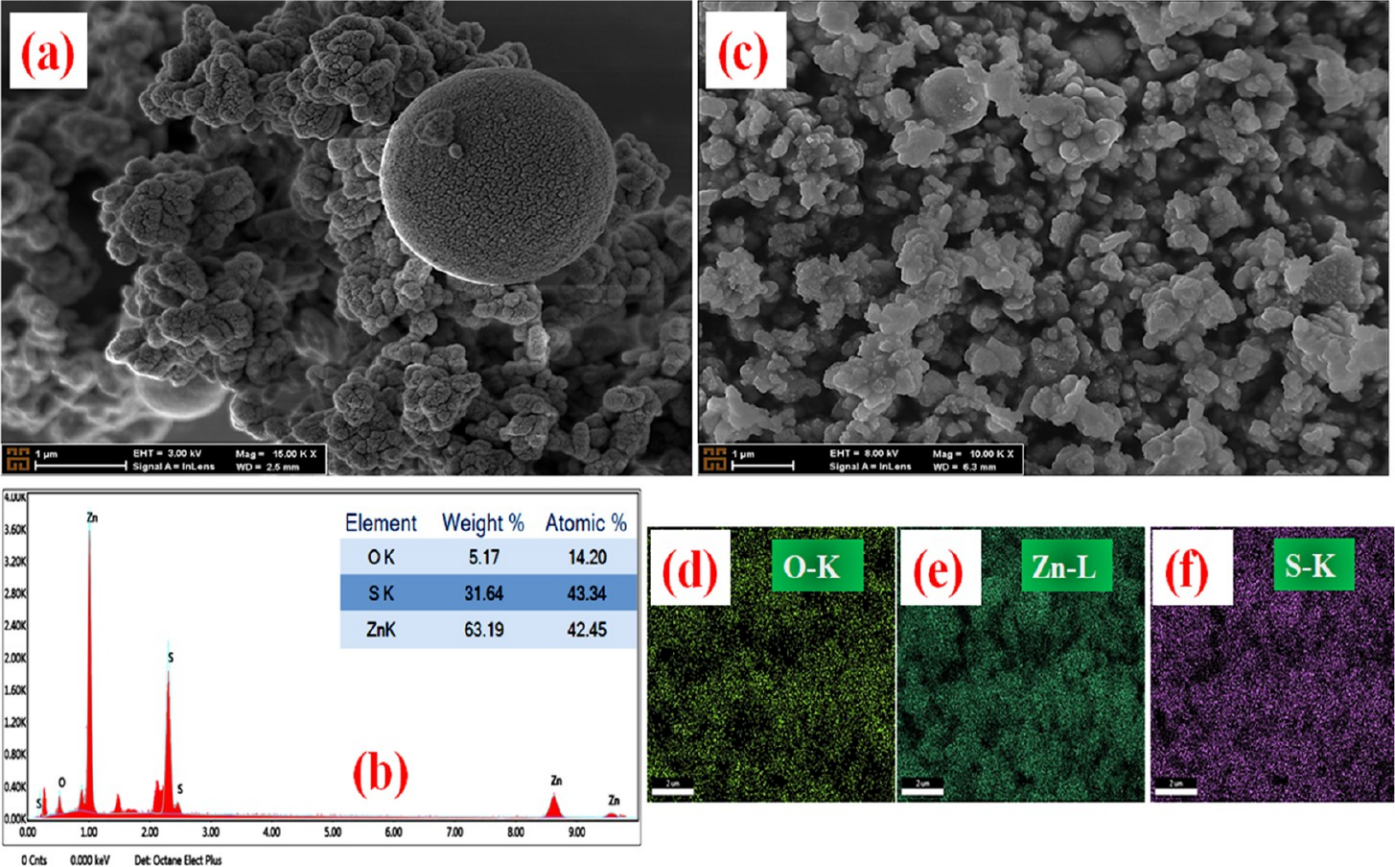
(a–c) FE-SEM images, (b) EDX elemental analysis, and (d–f) mapping for morphological characterization with different magnifications for ZnS@ZnO.

However, quasi-spherical forms with sizes of 50–100 nm are also seen in its structure. FE-SEM images of the ZnS@ZnO composite structure (Figure 3a–f) obtained by the experimental processes shown schematically in Supporting Information Figure S1 show that the particles generally form an agglomerate structure together with spherical forms with diameters around 400 nm.

**Figure 4:**
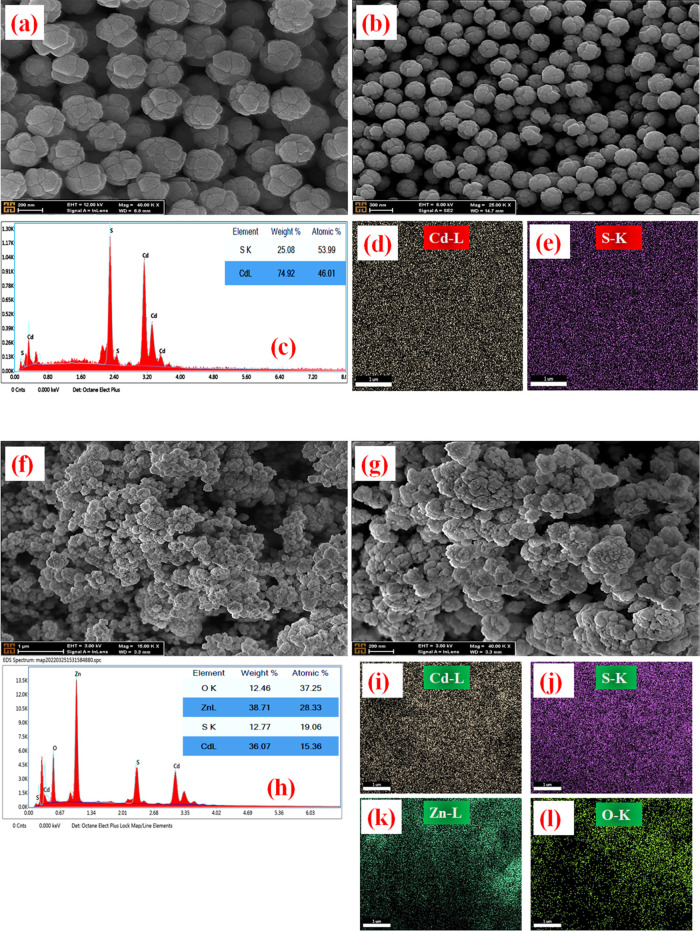
FE-SEM images, EDX elemental analysis, and mapping for morphological characterization with different magnifications for CdS (a–e) and CdS@ZnO (f–l).

The morphological structures of CdS and CdS@ZnO are presented in Figure 4. Moreover, EDX analysis and X-ray mapping reflected only the presence of cadmium (Cd), sulfur (S), zinc (Zn), and oxygen (O) elements in the spectrum, and no other elements were detected. The CdS is clearly seen to consist of submicrospheres with diameters less than 400 nm and were almost uniform in size although they showed a small amount of aggregation form (Figure 4a–b). The surface structure of CdS@ZnO (Figure 4f–g) appeared to be more agglomerated compared to CdS. It is also possible to encounter quasi-spherical formations smaller than 50 nm in size within the structure.

**Figure 5:**
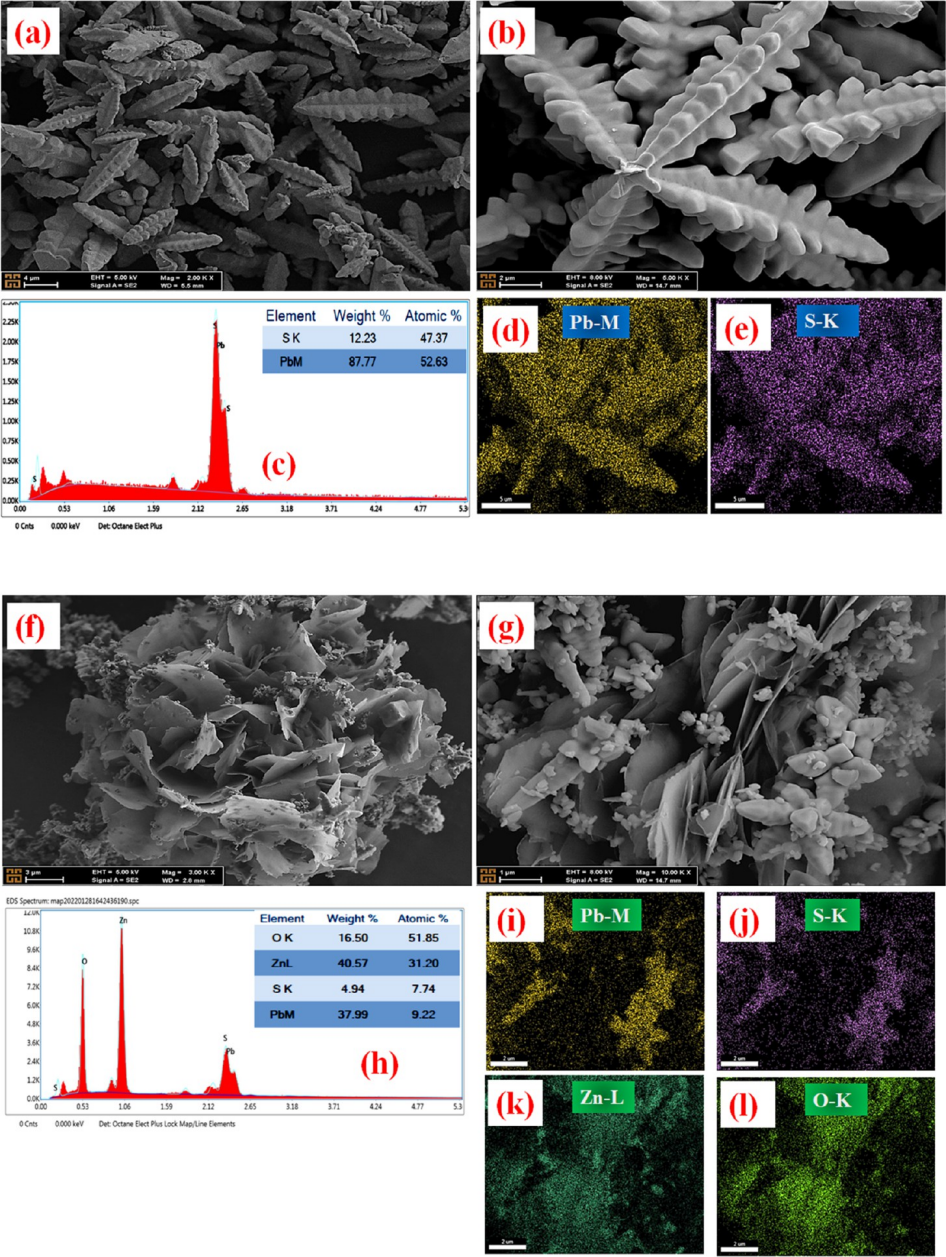
FE-SEM images, EDX elemental analysis, and mapping for morphological characterization with different magnifications for PbS (a–e) and PbS@ZnO (f–l).

Figure 5a,b shows the SEM images obtained for PbS semiconductor materials. From these images, it was observed that the PbS surface structure was a dendrite-like morphology with a length of ∼2–3 μm. In addition to the EDX spectrum and X-ray map revealing the presence of both Pb and S elements, quantitative data in the element tables confirm the presence of compositional elements of the sample. When the X-ray mapping results of the composite PbS@ZnO are examined (Figure 5i–l), it can be seen that a morphology with flower-like flake ZnO structures and PbS structures scattered among them emerges. The purity of the prepared sample is understood from the absence of any contamination or impurity peak in the EDX spectrum. Moreover, the X-ray maps reveal that all of the present elements are almost homogeneously dispersed throughout the corresponding powder samples.

### 3.3 Raman Spectra and FTIR Analysis

**Figure 6:**
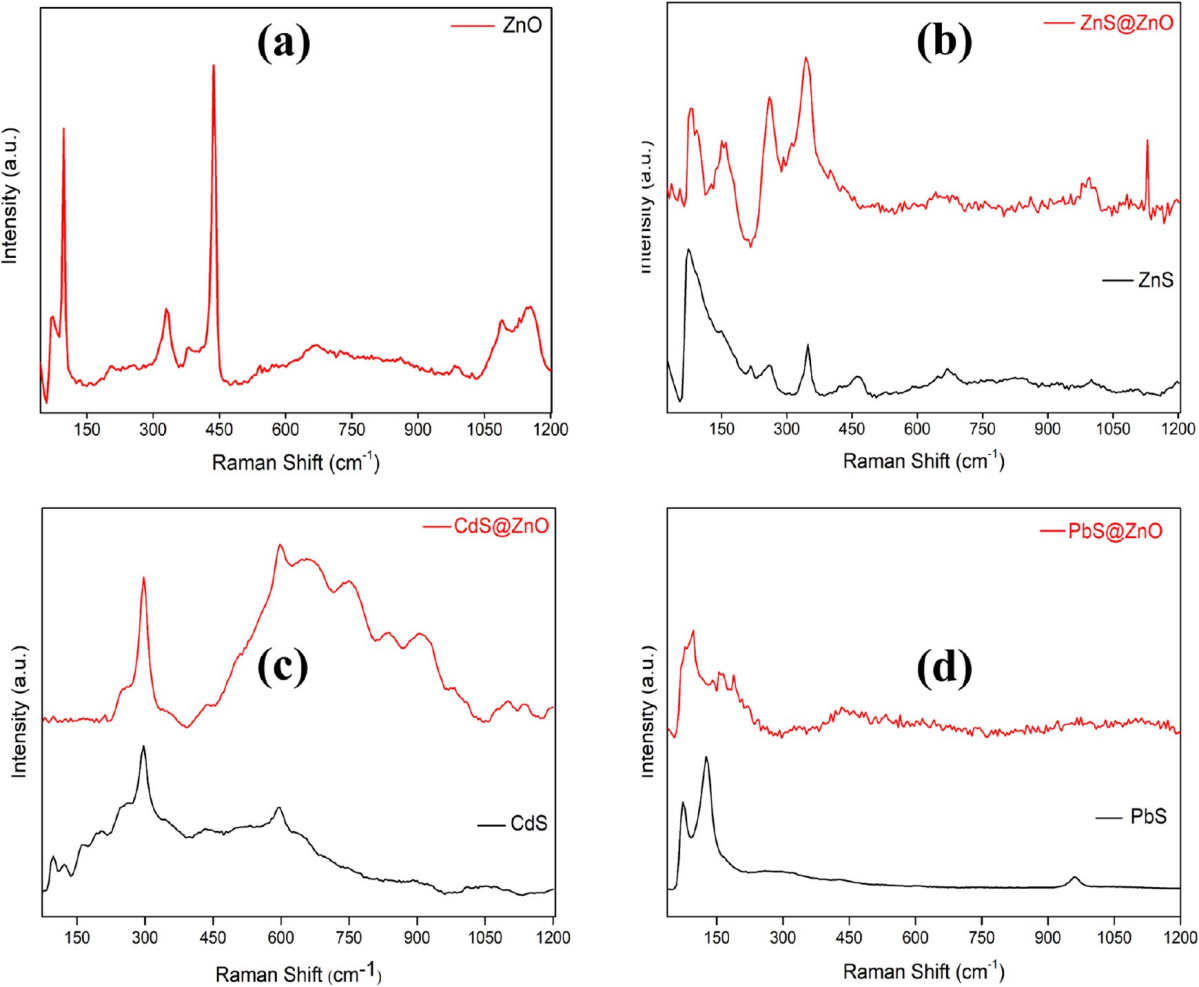
Raman spectra of ZnO (a), ZnS and ZnS@ZnO (b), CdS and CdS@ZnO (c), and PbS and PbS@ZnO (d).

In this study, the vibrational modes and phase structures of all powder samples were evaluated by examining the Raman spectra obtained with a confocal Raman spectrometer (Figure 6). The ZnO-Raman spectrum is shown in Figure 6a, including the low wavenumber region (LWR, 0–120 cm^–1^) and the high wavenumber region (HWR, 300–600 cm^–1^). Localized acoustic phonon modes are broad bands below 50 cm^–1^.^46^ The characteristic vibration band values corresponding to ZnO are 97 cm^–1^ (LWR-optical phonon E_2_ mode), 331 cm^–1^ (second-order spectrum of ZnO), 439 cm^–1^ (HWR-optical phonon E_2_ mode), and 1152 cm^–1^ (multiphonon scattering).^47^ The E_2_ (high) Raman mode detected at 439 cm^–1^ in this graph is mainly due to oxygen vibration.^48,49^

Figure 6b shows the highest Raman peaks for ZnS nanoparticles at 74, 259, 349, 1343, and 1572 cm^–1^, respectively, and other vibration peaks originated from the acetate peak at 352 cm^–1^ in the Raman spectrum of ZnS indicates the longitudinal optical (LO) modes of A_1_ and E_1_ symmetries,^51,52^ and finally, the peaks at 1343 and 1572 cm^–1^ can be attributed to multiphonon scattering group. The strong peak at 74 cm^–1^ is attributed to the LWR-optical phonon E_2_ mode,^50^ and the weak peak observed at 284 cm^–1^ represents the transverse HWR-optical (TO) phonon E_2_ mode.^50^

As seen in Figures 1 and 6, XRD results for ZnS@ZnO, CdS@ZnO, and PbS@ZnO samples indicated the formation of composites, and Raman spectra reflected the characteristics of the peaks of ZnO, ZnS, CdS, and PbS. Looking at the results from another perspective, it shows that the composite materials have a successful formation and good integration. Table 2 shows the Raman-active phonon frequencies (unit cm^–1^) in different wavenumber regions for the semiconductor photocatalysts obtained in this work.

**Table 2:** Raman-Active Phonon Frequencies (Unit cm^–1^) in Different Wavenumber Regions for Semiconductor Photocatalysts in This Work (tw)^a^

mode	ZnO	ZnS	CdS	PbS
E_2_ (LWR) (TW)	97	74		
E_2_ (LWR) [ref]	100^47^	72^50^	44^55^	
E_2_ (HGR) (TW)	439	284	257	
E_2_ (HGR) [ref]	438^47^	286^50^	252^55^	
A_1_-(TO) (TW)	377	266	216	131
A_1_-(TO) [ref]	380^36^	274^54^	212^56^	134^57^
E_1_-(TO) (TW)	408	266	230	131
E_1_-(TO) [ref]	407^36^	274^54^	245^56^	134^57^
A_1_-(LO) (TW)	570	352	301	216
A_1_-(LO) [ref]	574^50^	351^52^	305^56^	212^58^
E_1_-(LO) (TW)	581	352	301	216
E_1_-(LO) [ref]	584^53^	351^52^	305^56^	212^58^

a Modes A_1_ (TO, transverse optic) and E_1_ (LO, longitudinal optic) are polar modes and are both Raman and infrared active. E_2_ modes are nonpolar and only Raman active.

According to the FTIR results given in Supporting Information Figures S2 and S3, the FTIR peaks at 585 and 1082 cm^–1^ correspond to metal oxide stretching vibrations of Zn–O bonds. The metal–oxygen frequencies for the ZnO NPs are in accordance with literature.^59^ The FTIR peaks observed between 700 and 500 cm^–1^ correspond to the stretching of Zn–S bonds, which are in strong agreement with the reported results.^60^ For CdS NPs, a medium absorption peak at 669 cm^–1^ belongs to Cd–S bond stretching vibration.^61^ The FTIR peaks for the stretching of the Pb–S bond are obtained at 588, 1073, and 1443 cm^–1^.^62^

### 3.4 Optical Studies

The optical properties (absorption spectra and band gap) of all synthesized samples were examined using absorbance measurements obtained by UV–visible absorption spectroscopy over a spectrum ranging from 250 to 1100 nm.

**Figure 7:**
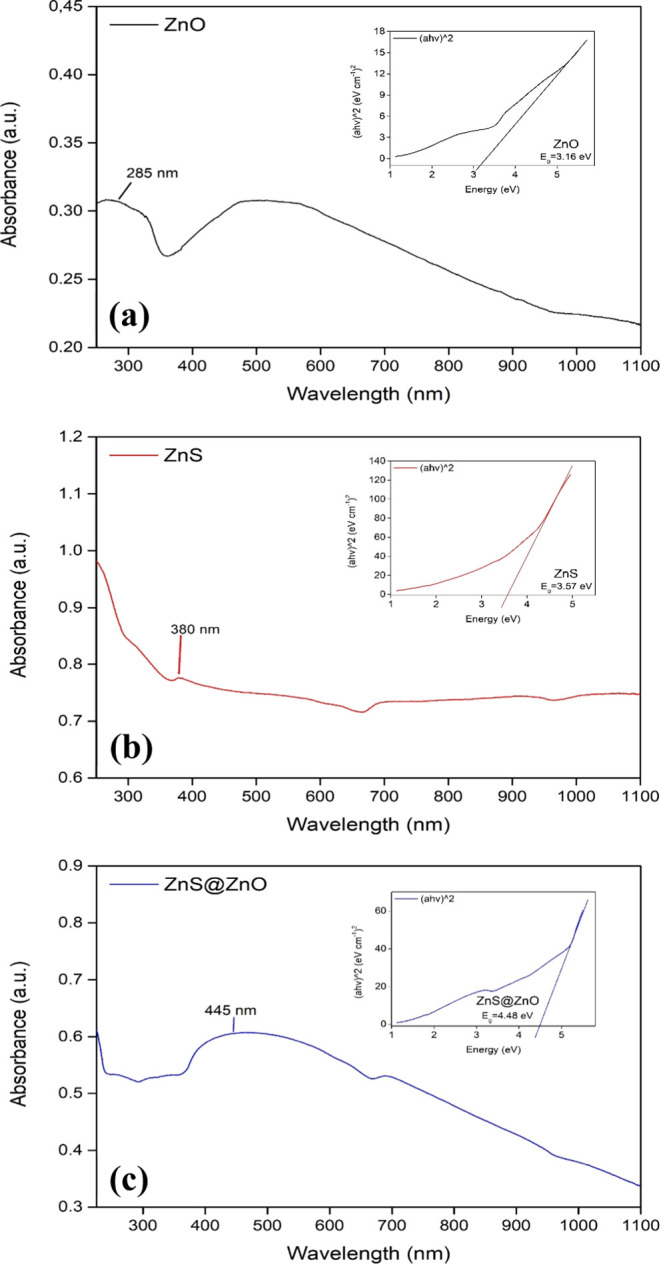
UV–Vis spectra and the corresponding Tauc plot of ZnO (a), ZnS (b), and ZnS@ZnO (c) semiconductor photocatalysts.

In the absorption spectra (Figure 7), the maximum absorption values for ZnO and ZnS appear around 285 and 380 nm wavelengths, respectively. The transfer of electrons (from the valence band to the conduction band) can be associated with the absorption edges of semiconductor photocatalysts.^63^ A maximum absorption peak centered around 445 nm was observed for the binary composite ZnS@ZnO, revealing different absorption property spectra from individual components, as expected. Consequently, this difference in the binary composite can be attributed to significant interfacial contact between the components and electronic transitions from the valence band to the conduction band. Additionally, the maximum absorption peaks for CdS, CdS@ZnO, PbS, and PbS@ZnO were observed to be around 332, 401, 391, and 450 nm wavelengths, respectively (Supporting Information Figure S4).

The band gap of the material can be determined by the fundamental absorption corresponding to the transition from the valence band to the conduction band. The plot of (α*h*ν)^2^ versus photon energy (*h*ν) is used for the direct band gap energy determination. The following relationship was used to estimate the optical band gap energies (*E*_g_) of all pure and composite samples in this study
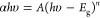
9where ν is the frequency, *h* is Planck’s constant, *A* is a constant that has a value close to 1 and energy independent, α is the absorption coefficient, and *n* = 1/2 for the allowed direct band gap.^64^

Using Tauc’s plots (embedded graphics in Figure 7) adopted to estimate the band gap values, the direct band gap values for ZnO, ZnS, and ZnS@ZnO were noted as 3.16, 3.57, and 4.48 eV, respectively. These values were determined by extrapolating the linear portions of the curves near the onset of the absorption edge to the energy axis. The values of 3.16 and 3.57 eV obtained for ZnO and ZnS are remarkable in that they are in good agreement with the data of 3.23^65^ and 3.68^11^ presented in the literature. The data here also confirm the intended heterojunction formation, with the aim of enabling the operation of the photocatalysis mechanism in the UV–visible light range. Owing to the high absorption of the ZnS@ZnO heterojunction in the visible light range, the active interfacial interaction between ZnS and ZnO causes the effective separation of electrons–holes. Hereby, visible light absorption in the solar spectrum is used efficiently and leads to improved photoelectrocatalytic activity. Lastly, using eq 7, the band gap values of 2.55, 4.81, 2.11, and 4.93 eV obtained for CdS, CdS@ZnO, PbS, and PbS@ZnO, respectively, agree with the literature values of 2.40 eV for CdS^12^ and 2.0 eV for PbS^21^ (Supporting Information Figure S4).

### 3.5 Photocatalytic Degradation-Based Removal Studies

**Figure 8:**
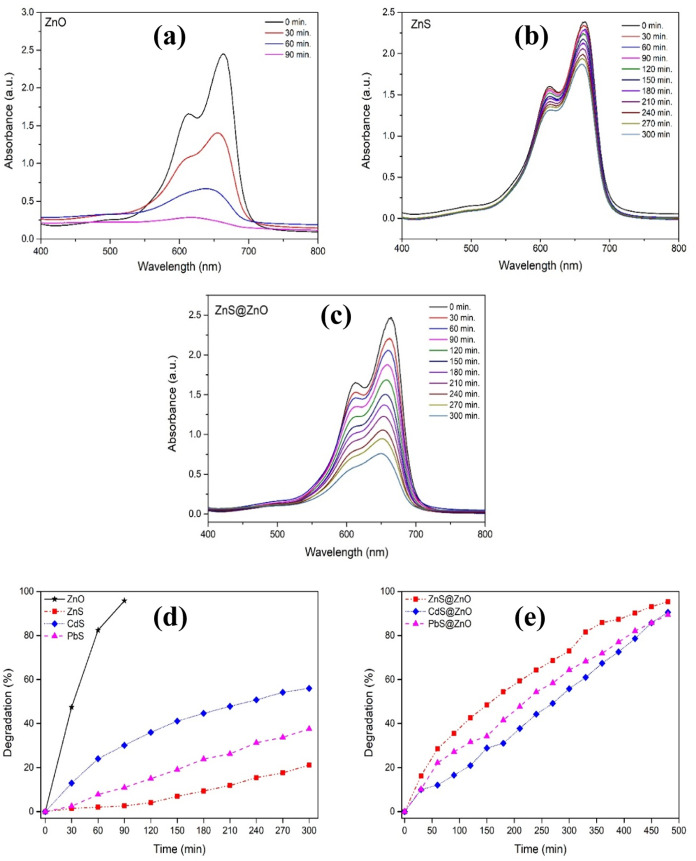
Time-dependent UV–vis spectra of 10 ppm solution of methylene blue (MB) dye using ZnO (a), ZnS (b), and ZnS@ZnO (c), respectively. Photocatalytic degradation (%) of MB dye on ZnO, ZnS, CdS, and PbS (d) and ZnS@ZnO, CdS@ZnO, and PbS@ZnO (e) as a function of time.

ZnO was reported to be even more effective than TiO_2_ in degrading organic pollutants in aqueous systems.^66^ In addition, the fact that ZnO is cheaper than TiO_2_ makes it an economically suitable photocatalyst for large-scale water purification systems. In some recent studies in the literature,^67−75^ it is seen that nanocatalysts obtained as a result of modification of ZnO with metals and metal oxides are used for the photodegradation and decolorization of methylene blue (MB-C_16_H_18_ClN_3_S) dye to reveal this effect. Therefore, in this study, to investigate the photocatalytic effect of heterojunction composites formed by ZnO and pure metal sulfides, the photocatalytic activity of pure ZnO, ZnS, CdS, PbS, and heterojunction composites was examined by degradation of MB dye, an organic pollutant, under UV light (400 W halogen lamp). It is known that the characteristic absorption peaks of MB occur at wavelengths centered at 614 and 664 nm at the end of the photodegradation mechanism.^76^ According to Figure 8, the time-dependent UV–vis spectra of 10 ppm of MB dye using ZnO, ZnS, and ZnS@ZnO in this study similarly also showed that MB has two absorption maximums. Moreover, Figure 8a–c depicts that in the irradiation time range of 0–300 min, the characteristic absorbance maximum intensity of MB decreases.

In this study, among all semiconductor photocatalysts synthesized by the hydrothermal method, ZnO showed the highest photodegradation efficiency with a value of 95.8% (eq 1) in a short time of 90 min, as expected (Figure 8d). The degradation rates (%) were 56.1, 37.5, and 21.2% for pure metal sulfides CdS, PbS, and ZnS in the 0–300 min time range (Figure 8d), while 95.3, 90.5, and 89.4% were obtained for heterojunction composites ZnS@ZnO, CdS@ZnO, and PbS@ZnO in the 0–480 min time range (Figure 8e), respectively. It is reported that the basis of photocatalytic mechanisms is the efficient production and separation of carriers by band gap excitation during the process.^77^ Based on this, the most important factor affecting the photocatalytic reaction is the photoinduced formation and separation of electron–hole pairs.^78^

During the pure ZnS photocatalytic reaction, a small fraction of available holes and electrons that can participate in the photocatalytic reaction might occur due to the rapid recombination of charge carriers.^79^ Therefore, pure ZnS samples have a lower photocatalytic activity than CdS and PbS. As for the heterojunction nanocomposites, the change in degradation after 300 min for CdS@ZnO was too small to be noticed (55.9%). The value of 64.3% obtained for the degradation rate for PbS@ZnO increased by approximately 70% compared to pure PbS. However, the findings showed a remarkable increase in the degradation of MB dye in the presence of the ZnS@ZnO heterojunction nanocomposite after 300 min of degradation (72.9%).

**Figure 9:**
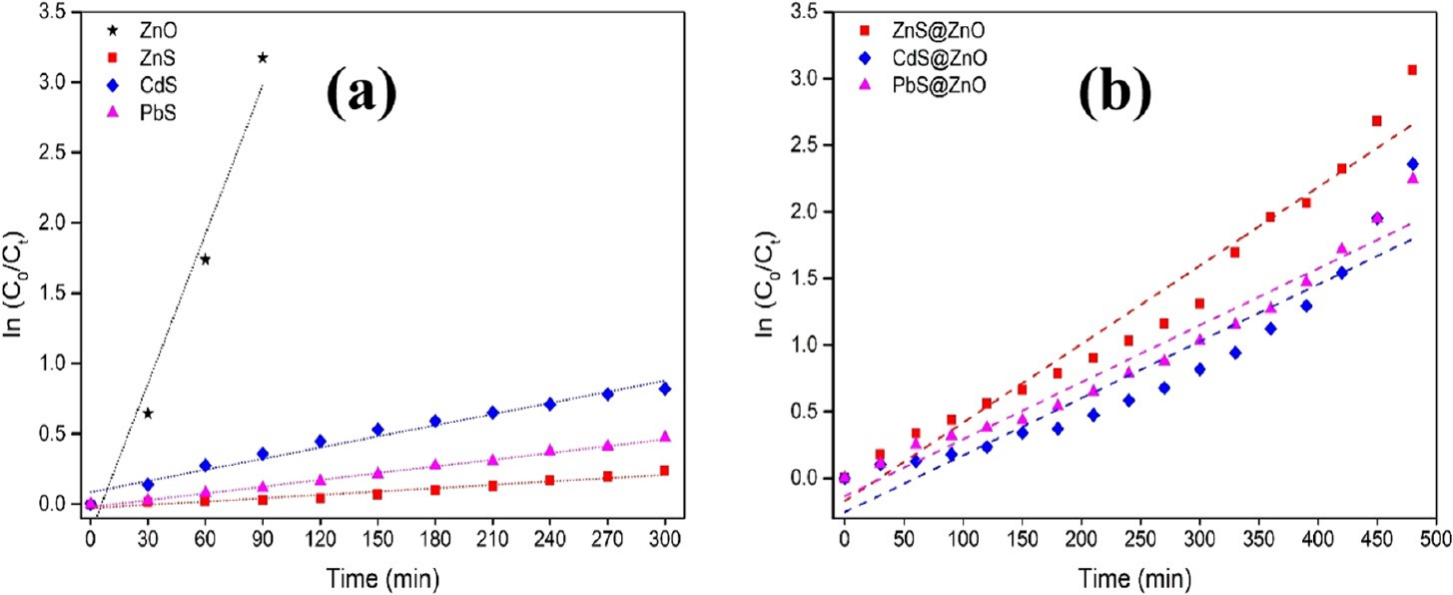
Plots of photocatalytic activity of MB on ZnO, ZnS, CdS, and PbS (a) and ZnS@ZnO, CdS@ZnO, and PbS@ZnO (b) as a function of time, respectively ln(*C*_0_/*C_t_*).

Using the pseudo-first-order chemical kinetics reaction given in eq 2 (Langmuir–Hinshelwood equation model), the catalytic performance of all semiconductor photocatalysts was evaluated and is depicted in Figure 9. The plot of ln(*C*_0_/*C_t_*) as a function of time for all samples was presented as a linear regression with a slope equivalent to the apparent rate constant *k* (min^–1^). Also, the half-life times (*t*_1/2_) were calculated by using eq 5. From the calculations, it was found that the rate constant and half-life time values for pure ZnO, ZnS, CdS, and PbS were 0.0354 min^–1^ and 19.6 min, 0.0008 min^–1^ and 866.3 min, 0.0026 min^–1^ and 266.5 min, and 0.0016 min^–1^ and 433.1 min, respectively. Based on the results here (Figure 9a), we can say that they confirm the expectation that photodegradation rates would be both faster for ZnO and considerably slower for ZnS. From the data shown in Figure 9b and obtained in the 0–480 min time range to observe almost complete decolorization and degradation of the MB dye, the rate constant *k* (min^–1^) and the half-life time (*t*_1/2_) values for ZnS@ZnO, CdS@ZnO, and PbS@ZnO were calculated as 0.0059 min^–1^ and 117.5 min, 0.0043 min^–1^ and 161.2 min, and 0.0042 min^–1^ and 165.0 min, respectively. Based on the results obtained from all data and graphs, ZnS@ZnO is the most effective in the degradation of MB organic pollutants. The ZnS@ZnO obtained by combining ZnO and ZnS to both obtain heterojunction composite and increase the photocatalytic effect shows that it has improved photocatalytic activity due to efficient separation of charge carriers compared to pure ZnS.

To examine the photocatalytic reaction process, the proposed mechanism scheme for ZnS@ZnO, CdS@ZnO, and PbS@ZnO heterojunction nanocomposites is shown in Supporting Information Figures S5, S6, and S7. The ZnS, one of the II–VI group semiconductors, is in the class of materials used in promising applications in photocatalytic studies. The conduction band of ZnS is −1.47 eV and the band gap is ∼3.60 eV. This is more negative than the potential for reduction of H^+^ to H_2_.^80^ To accelerate the separation of photogenerated e^–^/h^+^, it is proposed to integrate ZnS with other materials in the process of the formation of a heterostructure. Zinc oxide (ZnO), which contains the element Zn and shows considerably good photocatalytic activity, has a relatively more positive conduction band position (−0.31 eV) compared to ZnS, with a band gap of ∼3.20 eV. Therefore, the matched energy band structure of the combination of ZnS with ZnO (ZnS@ZnO) helps to both improve the degradation activity of organic pollutants and accelerate the separation of e^–^/h^+^ (Figure S5).

Before the photocatalytic reaction, due to the interaction between the catalyst and the dye, the dye is adsorbed to the catalyst surface for approximately 24 h in the dark. Once the ZnS@ZnO heterojunction photocatalyst is irradiated with UV light with energy higher than the band gap of ZnO, this process begins with the excitation of electrons (e^–^) from the valence band (VB) to the conduction band (CB). Thereby, simultaneously with the excitation of electrons, the same amount of photogenerated holes (h^+^) are located in the VB. The reaction of the holes (h^+^) in the VB with H_2_O results in the formation of H^+^ and OH^•^ ions, which are hydroxyl radicals. Super oxide anions (O_2_^–^) are formed by the absorption of electrons (e^–^) in the CB by oxygen molecules on the surface of the catalyst,^81^ and these anions lose electrons and form the super oxide anion radical (O_2_^•^). Then, the reaction of O_2_^•^ with H^+^ forms H_2_O_2_ (hydrogen peroxide), which produces the most highly reactive hydroxyl radicals. Free hydroxyl radical (OH^•^) is formed by the decomposition of H_2_O_2,_^82,83^ and the highly reactive O_2_^•^ and OH^•^ radicals react with the MB dye, resulting in harmless, nontoxic byproducts.^84−86^ The whole photocatalytic reaction process (R1–R7) can be summarized as follows
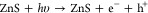
R1
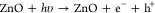
R2
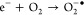
R3
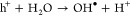
R4
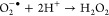
R5
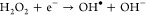
R6
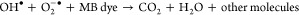
R7

The photocatalytic removal rates of the heterojunction photocatalysts used in this study are presented in Table S2, alongside the results of previous studies^87−95^ conducted under similar experimental conditions in the relevant field. According to the results in Table S2 and those obtained in this study, both the amount of catalyst and the irradiation time are important parameters that directly affect the system’s efficiency and the rate of photocatalytic reactions. An insufficient amount of catalyst limits the photocatalytic activity, while an excessive amount may prevent light from reaching the catalyst. Inadequate irradiation time can result in incomplete reactions, whereas excessive irradiation can reduce the catalyst’s efficiency and decrease the reaction yield. The results for different materials in Table S2 show that these two parameters vary depending on the material properties (such as catalyst type, morphology, surface area, etc.) and specific reaction conditions.

### 3.6 Determination of Optimum Catalyst Amount and Reusability Properties

**Figure 10:**
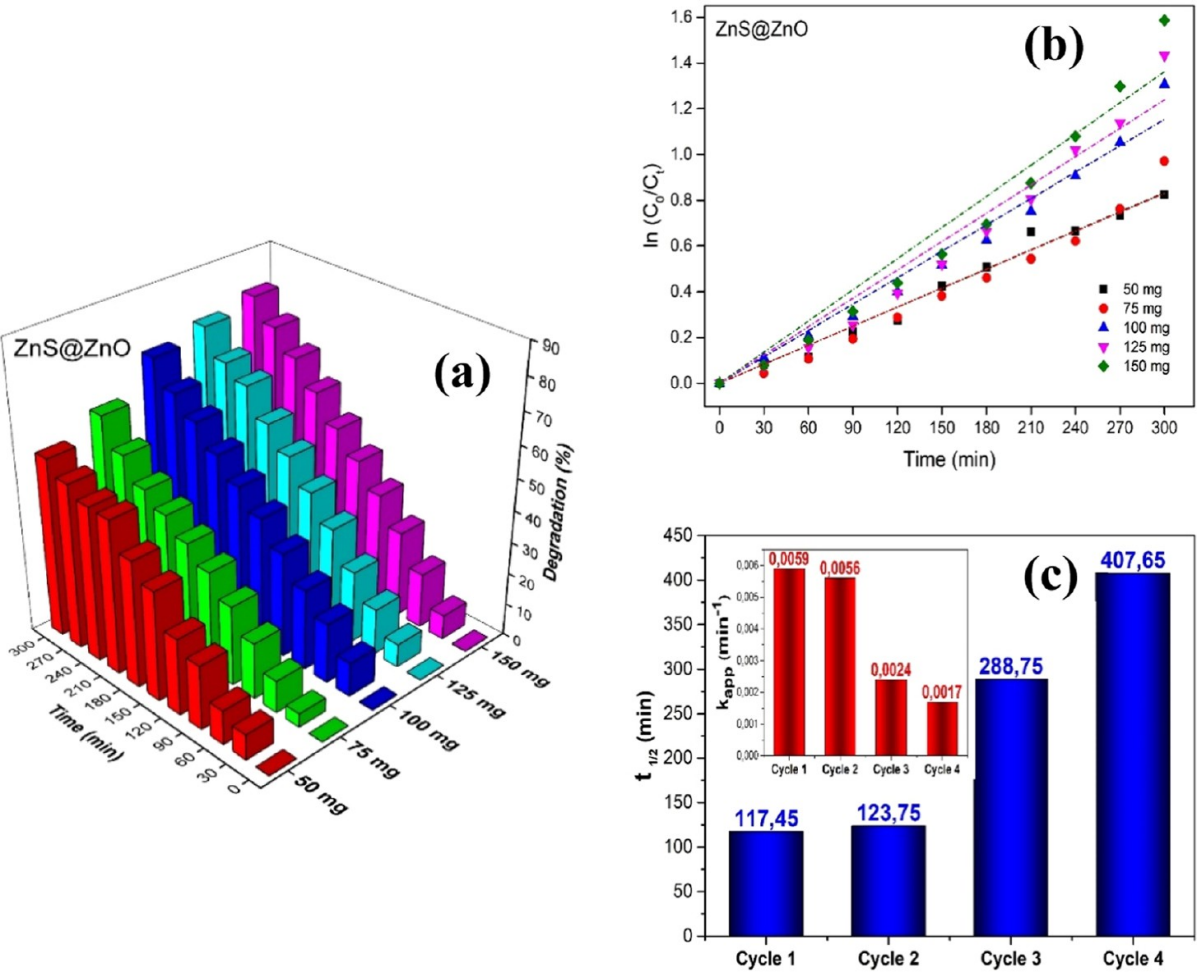
Photocatalytic degradation (%) of MB on ZnS@ZnO as a function of time for different amounts of substance (a) and the kinetic plot of ln(*C*_0_/*C_t_*) (b). *k*_app_ (apparent reaction rate constant) and half-life time of the photocatalytic decolorization rate of the MB (c) (reusability study for ZnS@ZnO).

In this study, after concluding that the ZnS@ZnO heterojunction semiconductor photocatalyst was more effective than others in degrading MB dye, experiments were carried out under similar conditions to determine the optimum amount of photocatalyst that could degrade this dye more effectively. The effect of changing the catalyst amount on the dye degradation was examined by increasing the catalyst amount by 25 mg from 50 mg to 150 mg and keeping the dye concentration constant (10 ppm) (Figure 10). Degradation rates (%) for ZnS@ZnO heterojunction hybrid photocatalyst with substance amounts of 50, 75, 100, 125, and 150 mg as 56.2, 62.1, 73.0, 76.2, and 79.5% were obtained in the 0–300 min time range, respectively (Figure 10a). From the ln(*C*_0_/*C_t_*) plots obtained in accordance with the Langmuir–Hinshelwood equation model in Figure 10b, it was observed that the increase in the catalyst amount increased the degradation efficiency (40% in total). By increasing the efficiency of superoxide anions (O_2_^•^) and hydroxyl radicals (OH^•^), which take an active part in the photocatalysis reaction after electron–hole pairs (e^–^/h^+^) formation, more needed active areas can be obtained by increasing the catalyst amount sufficiently. On the other part, when the amount of added catalyst is above a particular limit, a screening effect occurs in the photocatalyst particles due to particle–particle interaction, which prevents photons from reaching the photocatalyst surface and reduces the photodegradation efficiency.^96,97^ In this study, the added amounts above 100 mg did not increase degradation rates at expected values, which may be associated with this effect. As a result, the optimum catalyst amount at which the MB dye degradation rate increased most effectively was determined as 100 mg (apparent rate constant *k*: (min^–1^), 0.0042 min^–1^, half-life time: (*t*_1/2_) 165.0 min for 100 mg ZnS@ZnO).

To investigate the stability of the ZnS@ZnO hybrid nanocomposite prepared in the optimum amount (100 mg) determined for the photocatalytic reaction, reusability performance experiments were carried out for four cycles under the same conditions (Figure 10c). In the process of recycling the photocatalyst for the next dye degradation cycle, the photocatalyst was first washed after each cycle, collected by centrifuge, and dried in the oven at 50 °C for 2 h. The degradation rate values of the recycled samples were obtained as 95.33, 92.26, 67.86, and 58.87% (Table 3). As seen in Figure 10c, the rate constant (min^–1^) and half-life (*t*_1/2_) values for each cycle in the 0–480 min time range were calculated to be 0.0059 min^–1^ and 117.45 min (Cycle 1), 0.0056 min^–1^ and 123.75 min (Cycle 2), 0.0024 min^–1^ and 288.75 min (Cycle 3), and 0.0017 min^–1^ and 407.65 min (Cycle 4). When the photocatalytic activities of the photocatalysts used in each cycle were compared with the fresh ZnS@ZnO sample, it was calculated that there was a reduction of approximately 38% at the end of the fourth cycle. This decrease in the apparent rate constant *k* (min^–1^) values of the ZnS@ZnO semiconductor heterojunction photocatalyst, which was used repeatedly four times in a quite long-time interval, indicates that the ability of the photocatalyst to decompose the MB dye into simple molecules is limited after the fourth cycle.

**Table 3:** Degradation Rate Values of Recycled Samples for Each Cycle (0–480 min)

	degradation (%)			
time (min)	cycle 1	cycle 2	cycle 3	cycle 4
0	0.00	0.00	0.00	0.00
30	16.29	10.44	3.89	16.64
60	28.54	19.41	9.00	22.30
90	35.55	28.36	13.73	26.10
120	42.64	37.32	20.78	33.48
150	48.43	48.01	24.78	36.02
180	54.40	57.51	29.42	40.22
210	59.38	66.23	33.60	41.10
240	64.28	74.78	37.73	41.81
270	68.71	74.85	41.87	42.49
300	73.04	80.71	45.87	45.56
330	81.57	82.72	49.77	50.87
360	85.91	82.79	54.28	52.94
390	87.31	86.54	58.12	55.76
420	90.20	88.96	61.76	56.80
450	93.13	92.13	64.63	57.47
480	95.33	92.26	67.86	58.87

### 3.7 Effect of the pH on Adsorption-Based Removal of MB

**Figure 11:**
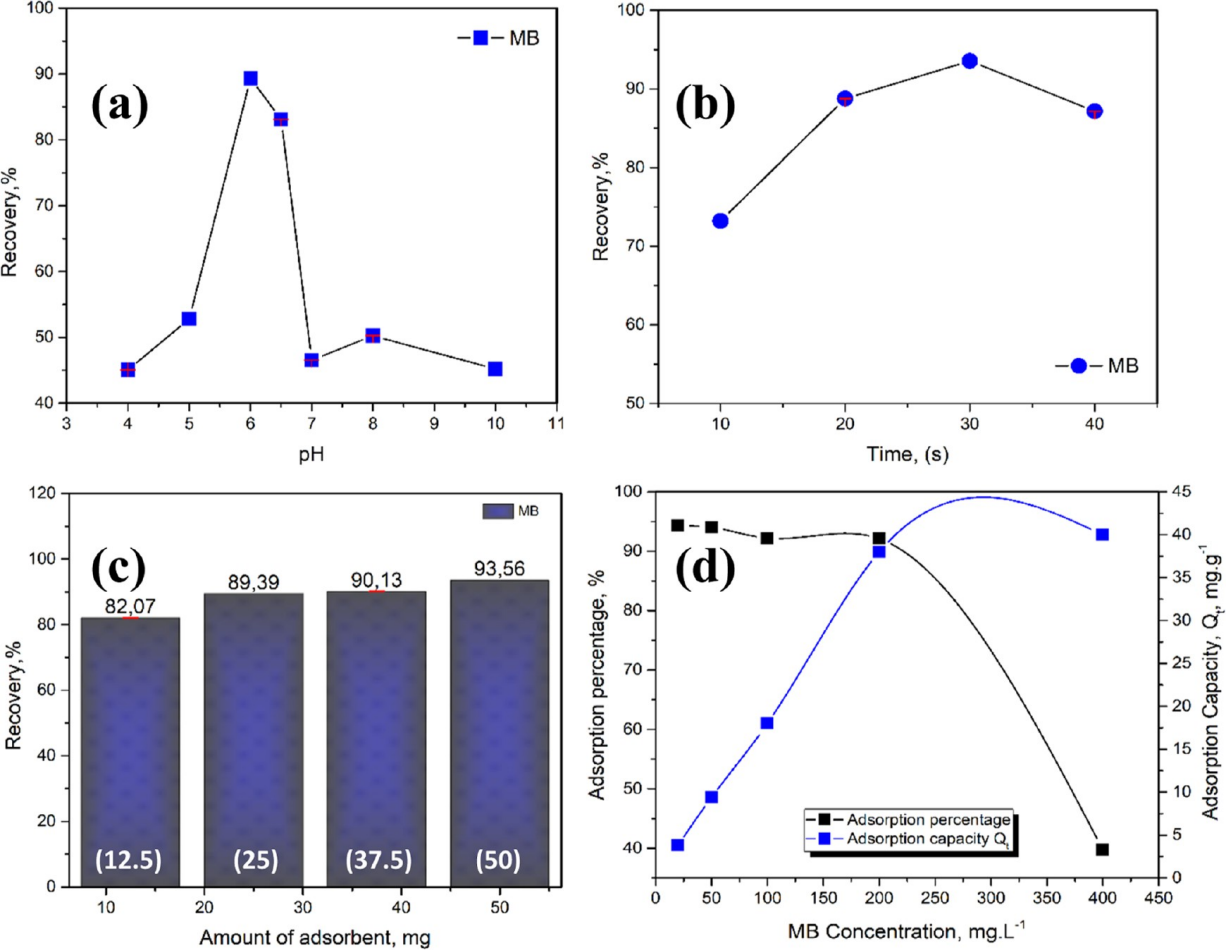
(a) Effect of pH on adsorption-based removal, (b) effect of contact time, (c) effect of the amount of ZnS@ZnO nanocomposite, and (d) adsorption graph of MB as a function of adsorption capacity and adsorption percentage of ZnS@ZnO nanocomposite (*N* = 3).

To evaluate the adsorption performance of the synthesized materials, MB was used as a representative model of organic pollutants. Figure 11a represents the change of MB adsorption percentage as a function of pH. Low adsorption percentages were observed for the ZnS@ZnO nanocomposite at pH 4.0, 5.0, 7.0, 8.0, and 10.0. The pH of the sample solution increases the adsorption performance at pH 6, where the performances are 45.1, 52.8, 89.3, 83.1, 46.5, 50.3, and 45.2% for pH 4.0, pH 5.0, pH 6.0, pH 6.5, pH 7.0, pH 8.0, pH 10.0, respectively. These changes were associated with electrostatic interactions between MB and the adsorbent. Since MB is present in cationic form in solutions, the acidity of the medium plays an important role in its retention. According to the observed results, pH 6 was determined as optimum for the adsorption-based removal of MB for ZnS@ZnO nanocomposite.

### 3.8 Effect of Contact Time on Adsorption-Based Removal of MB

During the adsorption process, MB continuously accumulated on the adsorbent, until the adsorbent was completely saturated. To evaluate the time required for maximum uptake of MB, the pH of the solution was fixed at pH 6 and the adsorbent mass at 50 mg, and the percentage of adsorption at different contact times was calculated (Figure 11b). At 10 min, it exhibited the lowest adsorption capacity due to low interaction with MB, while the maximum adsorption of MB was reached within 30 min. Therefore, 30 min was determined as the optimum contact time.

### 3.9 Effect of Mass on Adsorption-Based Removal of MB

The effect of the amount of ZnS@ZnO nanocomposite, between 12.5 and 50 mg, on the adsorption-based removal of MB was investigated (Figure 11c). The adsorption percentages of 12.5 25, 37.5, and 50 mg of adsorbent amount are 82.1, 89.4, 90.1, and 93.6%, respectively. The results showed that an increase in the amount of adsorbent per unit analyte concentration caused an increase in the efficiency of adsorption-based removal. Since the maximum adsorption efficiency was achieved with the addition of 50 mg of ZnS@ZnO nanocomposite, the optimum amount of adsorbent was determined as 50 mg.

### 3.10 Adsorption Capacity

The adsorption capacity was determined by the amount of substance adsorbed by the adsorbent. MB added at different concentrations was mixed for adsorption for 24 h. 1 mL of samples taken from the solution was analyzed with a UV–vis spectrophotometer. As a result of this analysis, the amount of adsorbed substance per unit mass was calculated, and the adsorption capacity was expressed as mg/g (milligram/gram). This optimized adsorption capacity increased the adsorption removal performance, enabling the more effective removal of unwanted pollutants from the environment. Figure 11d shows this more clearly graphically. The results show that the adsorption capacity at equilibrium increases with an increasing MB concentration and does not change after reaching 40 mg·g^–1^. On the other hand, the percentage of adsorption is higher at lower concentrations. This means that after a concentration of 200 mg·L^–1^ the adsorption sites of the material are completely filled by MB.

## 4 Conclusions

In this study, where the adsorption and photocatalytic properties of nanomaterials were combined on the same material, the removal efficiency of MB dye on ZnS@ZnO, CdS@ZnO, and PbS@ZnO nanocomposite materials was studied. Modification of ZnO nanorods with ZnS, CdS, and PbS nanoparticles with different morphologies gave the following results: (1) Hollow structures were formed by producing metal sulfide nanoparticles between ZnO nanorods to prevent agglomeration of ZnO nanorods, which leads to decrease adsorption and photocatalytic performance. (2) Creating gaps in the areas where metal sulfide and ZnO nanorods meet, where MB can hold, increased the adsorption efficiency. (3) By modification of the metal sulfide NPs (ZnS, CdS, and PbS) with ZnO nanorods, new materials with a lower band gap of metal sulfide nanoparticles were produced. As a result of the modification of metal sulfide NPs with ZnO nanorods, a new ZnS@ZnO nanocomposite could be applied to photocatalytic degradation of MB for at least 4 times and adsorption-based removal of MB for at least 10 times. The results proved that the new combined concept has the potential to be applied to large-scale wastewater treatment systems in the future.

## Supporting Information Available

Synthesis of semiconductor nanoparticles by hydrothermal synthesis method; the XRD results of powder samples; FTIR studies of all semiconductor nanoparticles; UV–Vis spectra and the corresponding Tauc plots of semiconductor photocatalysts; photocatalytic mechanism of dye degradation on the surface of the ZnS@ZnO, CdS@ZnO, and PbS@ZnO (PDF)
