# Supplementary material for: Comparison of Photocatalytic and Adsorption Properties of ZnS@ZnO, CdS@ZnO, and PbS@ZnO Nanocomposites to Select the Best Material for the Bifunctional Removal of Methylene Blue
Source: ACS Omega. 2025 Mar 4;10(10):9986–10003. doi: 10.1021/acsomega.4c07910 (PMC11923650; doi:10.1021/acsomega.4c07910)
Supplement: Supplementary file 1 — ao4c07910_si_001.pdf [file ao4c07910_si_001.pdf]

Supporting Information

Comparison of Photocatalytic and Adsorption Properties of ZnS@ZnO, CdS@ZnO and PbS@ZnO Nanocomposites to Select the Best Material for The Bifunctional Removal of Methylene Blue

*Ümit Bayram, Çiğdem Özer, Erkan Yılmaz**

Corresponding Author

Erkan Yilmaz − *Faculty of Pharmacy, Erciyes University, 38039 Kayseri, Turkey;*
Technology Research & Application Center (TAUM), Erciyes University, 38039 Kayseri, Turkey;
Erciyes University Nanotechnology Application and Research Center (ERNAM), 38039 Kayseri, Turkey;
Erciyes Teknopark ChemicaMed Chemical Inc., Erciyes University Technology Development Zone, 38039 Kayseri, Turkey; orcid.org/0000-0001-8962-3199;
Email: erkanyilmaz@erciyes.edu.tr

Authors

Ümit Bayram – *Central Research Facility (AGU-CRF), Abdullah Gül University, 38080, Kayseri, Turkey;*
Erciyes University Nanotechnology Application and Research Center (ERNAM), 38039 Kayseri, Turkey orcid.org/0000-0001-8760-8024

Çiğdem Özer – *Erciyes University Nanotechnology Application and Research Center (ERNAM),38039 Kayseri, Turkey*

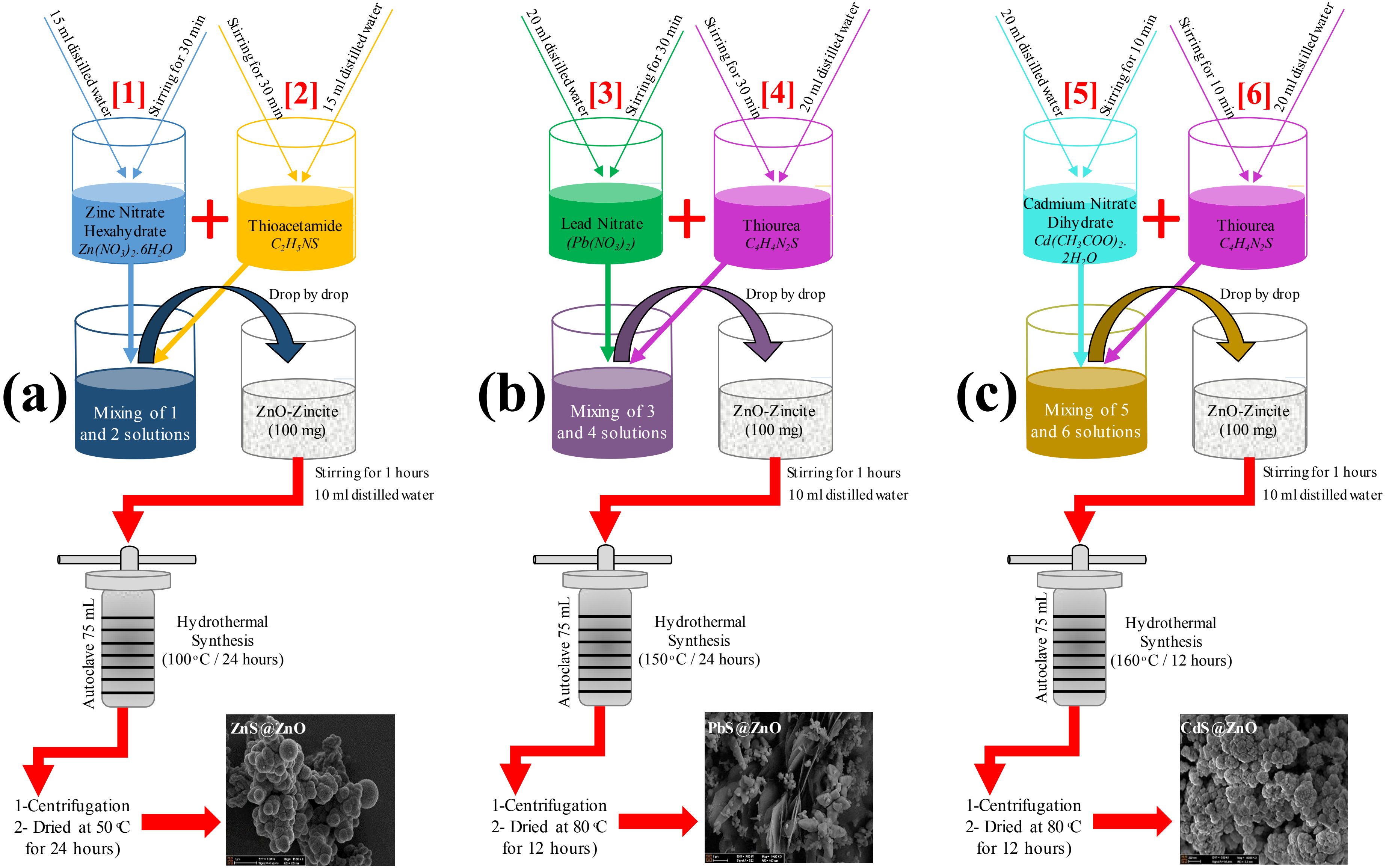

Figure S1. The schematic synthesis of ZnS@ZnO (a), PbS@ZnO (b) and CdS@ZnO semiconductor nanoparticles by hydrothermal synthesis method.

Table S1. The Results of XRD Analysis for ZnO, ZnS, CdS, And PbS Powder Samples.

Powder Sample	Miller indices (hkl)	Braggs angle (2θ) (deg)	d-spacing (Å)	Compound name (phase)	Compatible JCPDS Card no	Lattice parameter a (Å) and Space Group
ZnO	100	31.77	2.81	Zincite	No.36-1451	L.P. = 3.25 S.G. = P63mc (186) Hexagonal
	002	34.42	2.60			
	101	36.25	2.48			
	102	47.54	1.91			
	110	56.61	1.63			
	103	62.86	1.48			
	200	66.38	1.41			
	112	67.96	1.38			
	201	69.11	1.36			
	004	72.56	1.31			
	202	76.96	1.24			
ZnS	111	28.56	3.12	Sphalerite	No.05-0566	L.P. = 5.41 S.G. = F-43m (216) Cubic
	200	33.09	2.71			
	220	47.52	1.91			
	311	56.29	1.63			
	400	69.52	1.35			
	331	76.81	1.24			
CdS	100	24.81	3.59	Greenockite	No.41-1049	L.P. = 4.14 S.G. = P63mc (186) Hexagonal
	002	26.51	3.36			
	101	28.18	3.16			
	110	43.68	2.07			
	103	47.84	1.89			
	112	51.83	1.76			
	201	52.79	1.73			
	004	54.59	1.68			
	203	66.77	1.40			
	210	69.27	1.36			
	211	70.86	1.33			
	114	72.38	1.30			
	105	75.48	1.26			
PbS	111	26.01	3.42	Galena	No.65-9496	L.P. = 5.93 S.G. = Fm-3m (225) Cubic
	200	30.12	2.97			
	220	43.12	2.10			
	311	51.05	1.79			
	222	53.49	1.71			
	400	62.62	1.48			
	331	68.99	1.36			
	420	71.05	1.33			
	422	79.06	1.21			

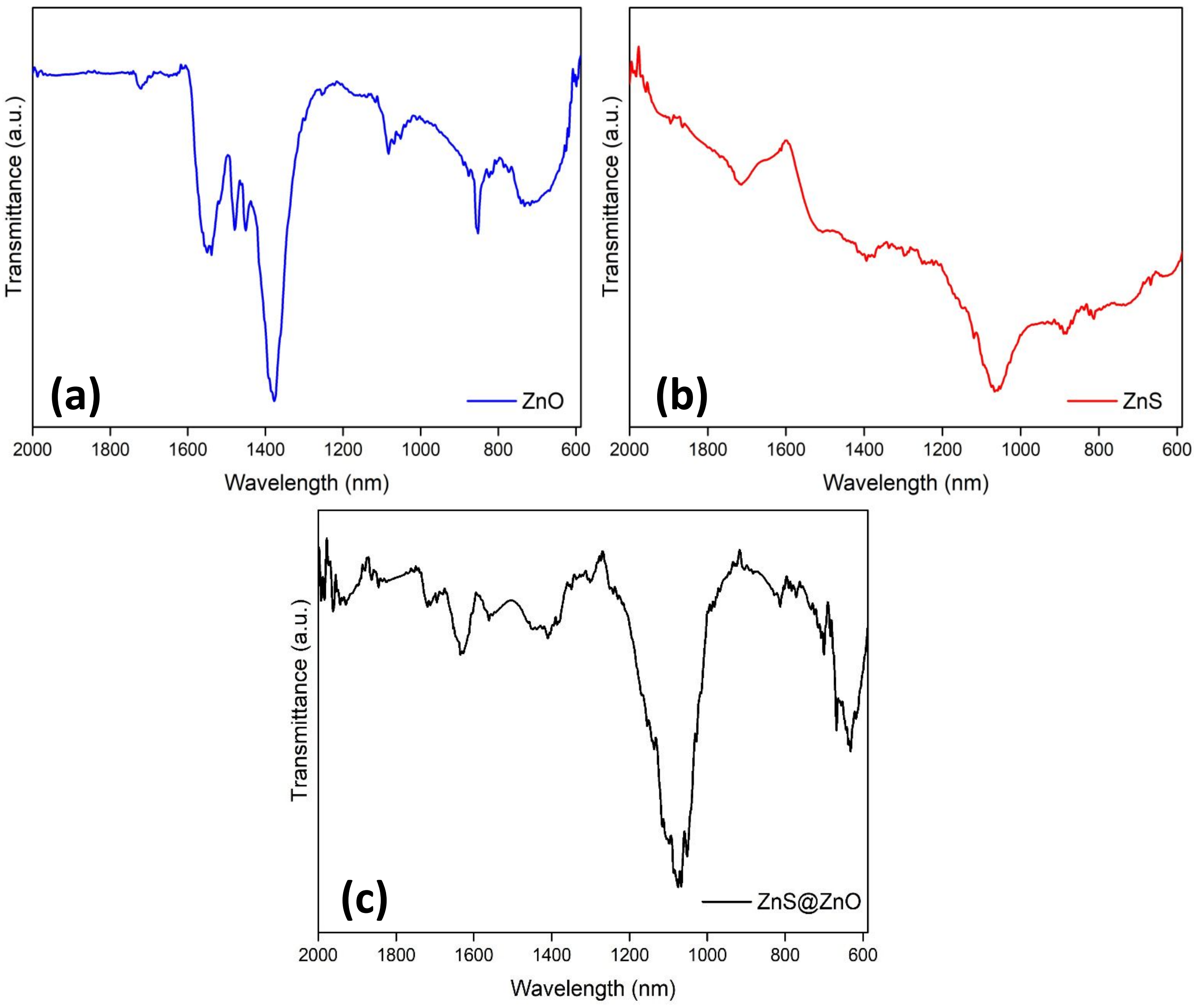

Figure S2. FT-IR studies of ZnO (a), ZnS (b), and ZnS@ZnO (c).

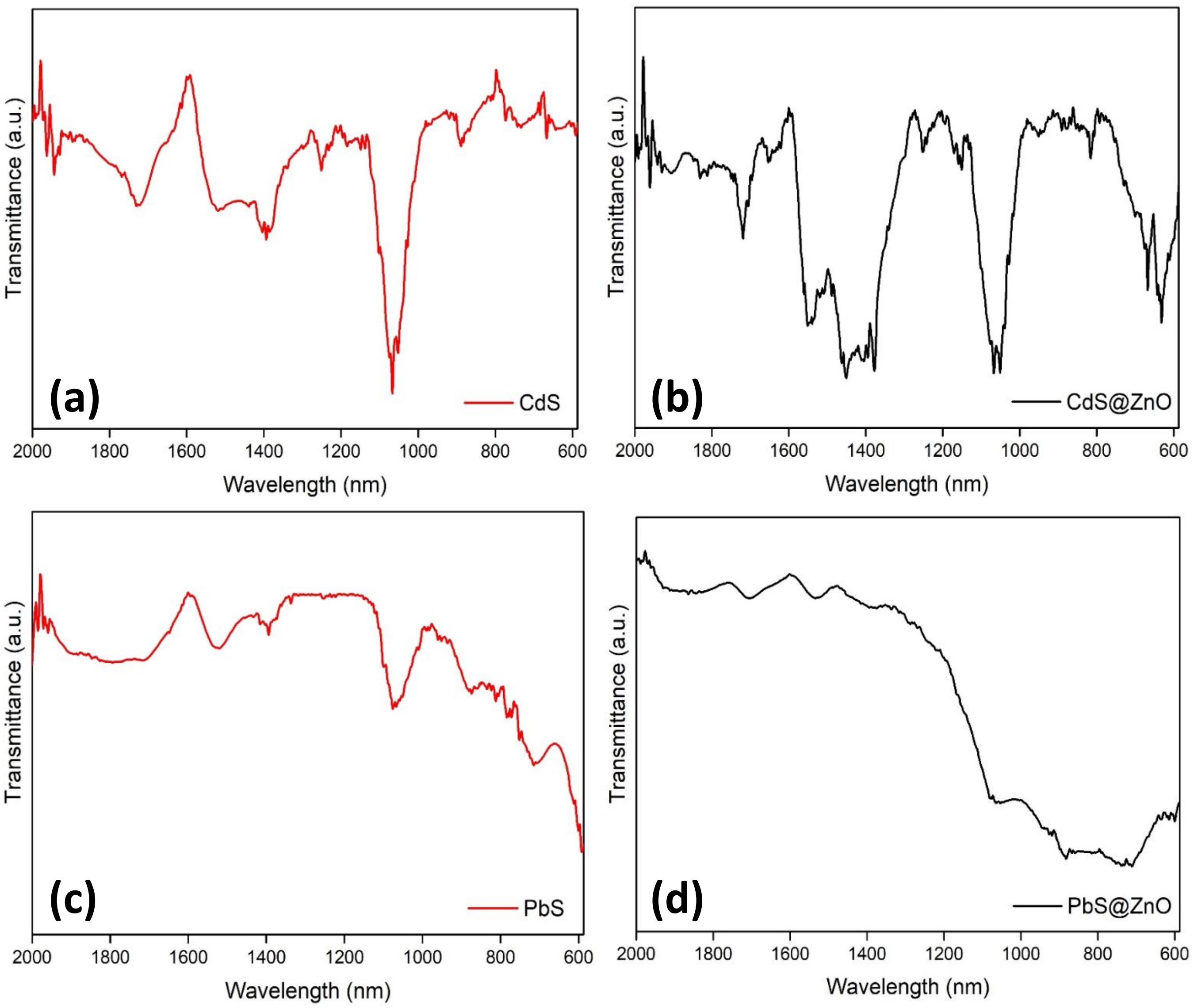

Figure S3. FT-IR studies of CdS (a), CdS@ZnO (b), PbS (c) and PbS@ZnO (d).

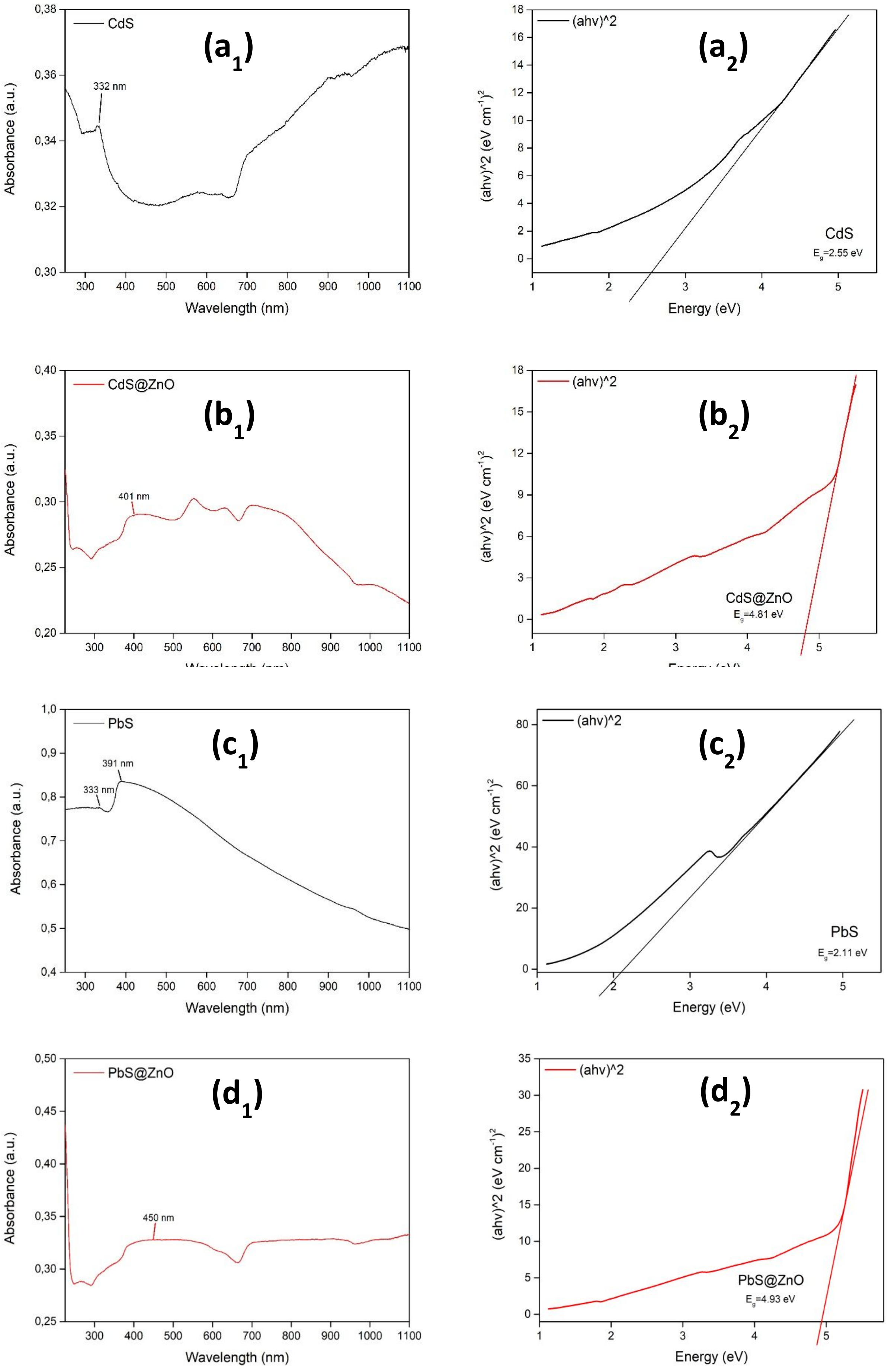

Figure S4. UV−Vis spectra and the corresponding Tauc plot of CdS (a_1-a_2), CdS@ZnO (b_1-b_2), PbS (c_1-c_2), and PbS@ZnO (d_1-d_2) semiconductor photocatalysts.

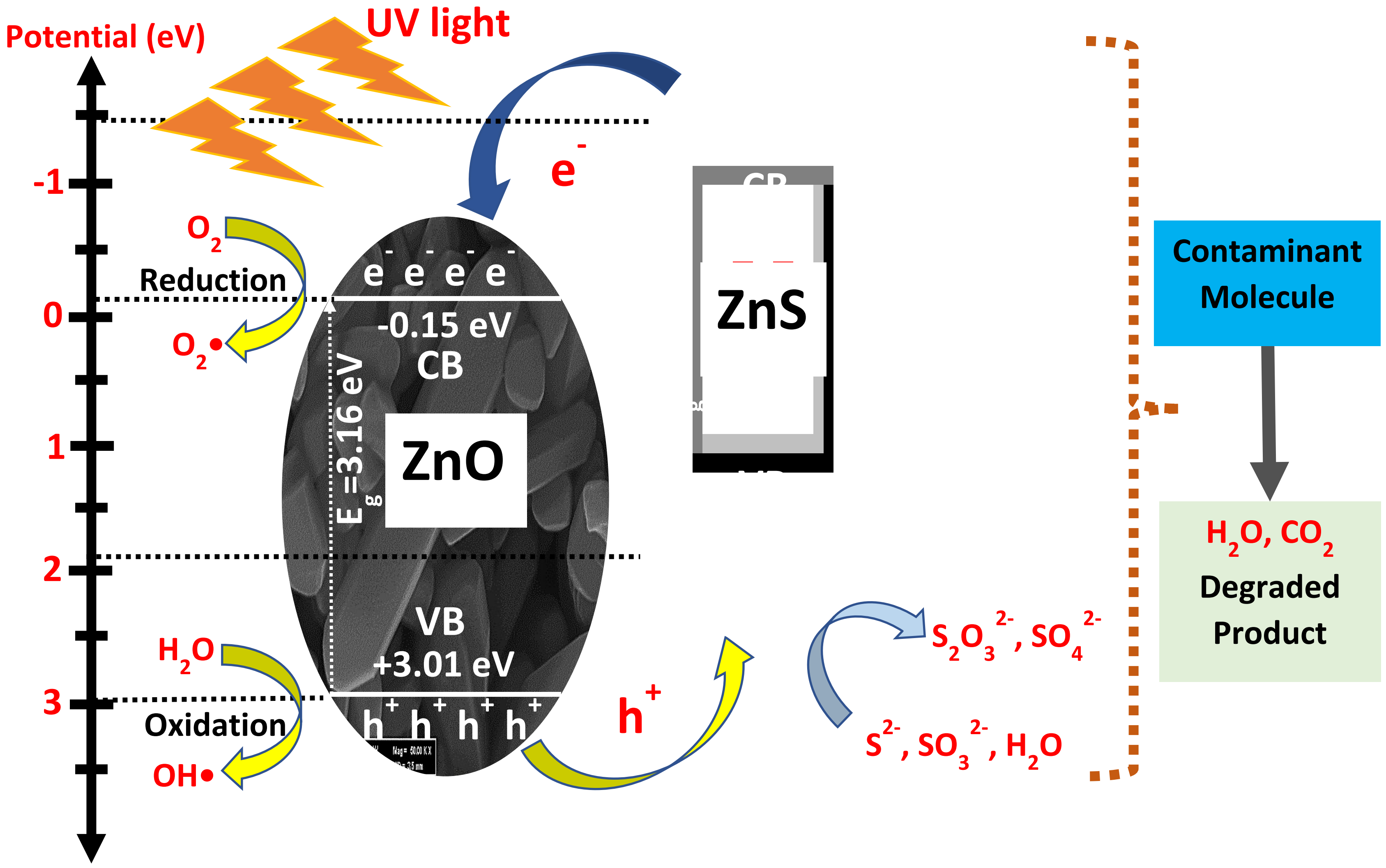

Figure S5. A proposed photo-catalytic mechanism of dye degradation on the surface of the ZnS@ZnO heterojunction semiconductor photocatalyst..

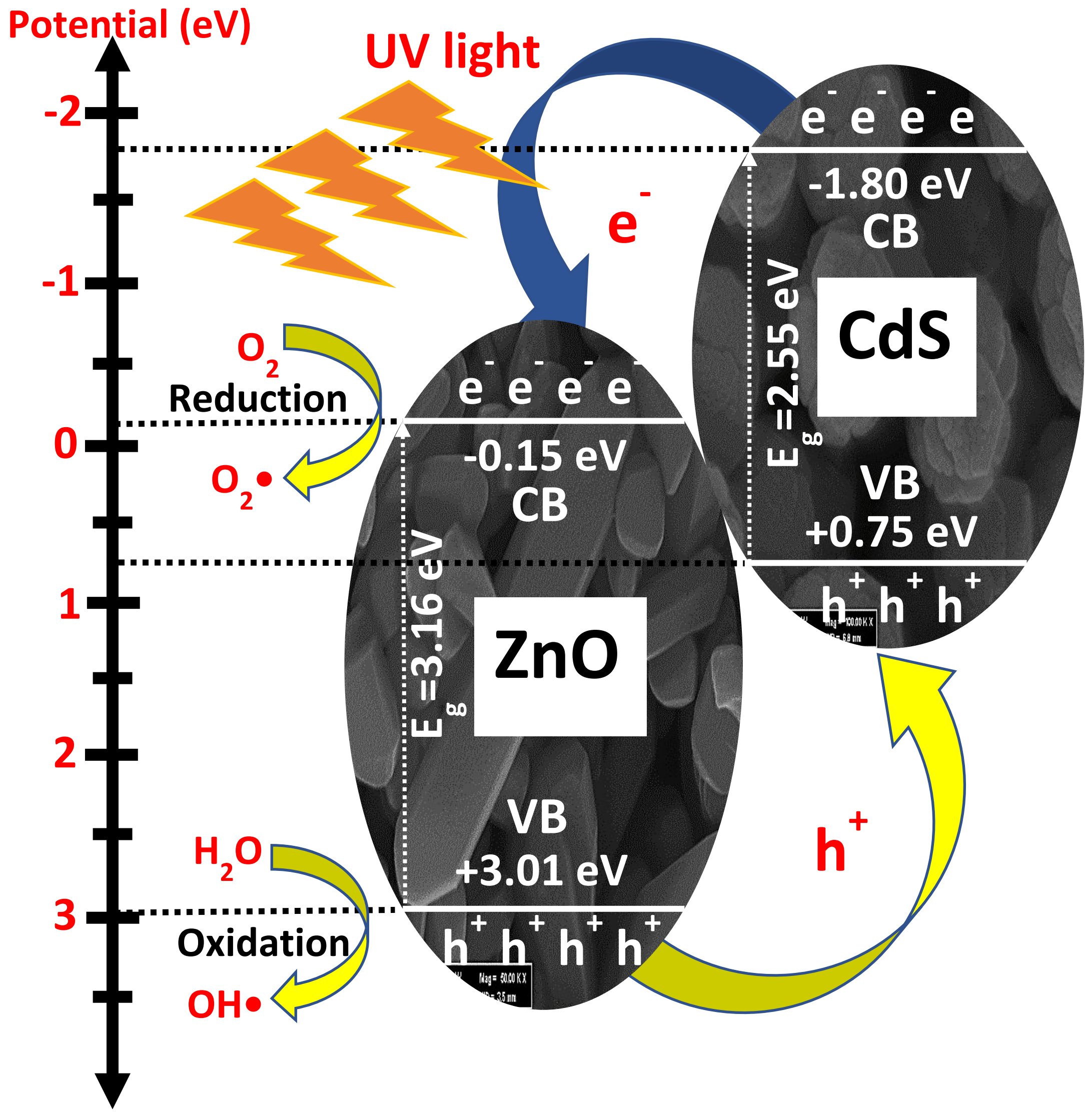

Figure S6. A proposed photo-catalytic mechanism of dye degradation on the surface of the CdS@ZnO heterojunction semiconductor photocatalyst.

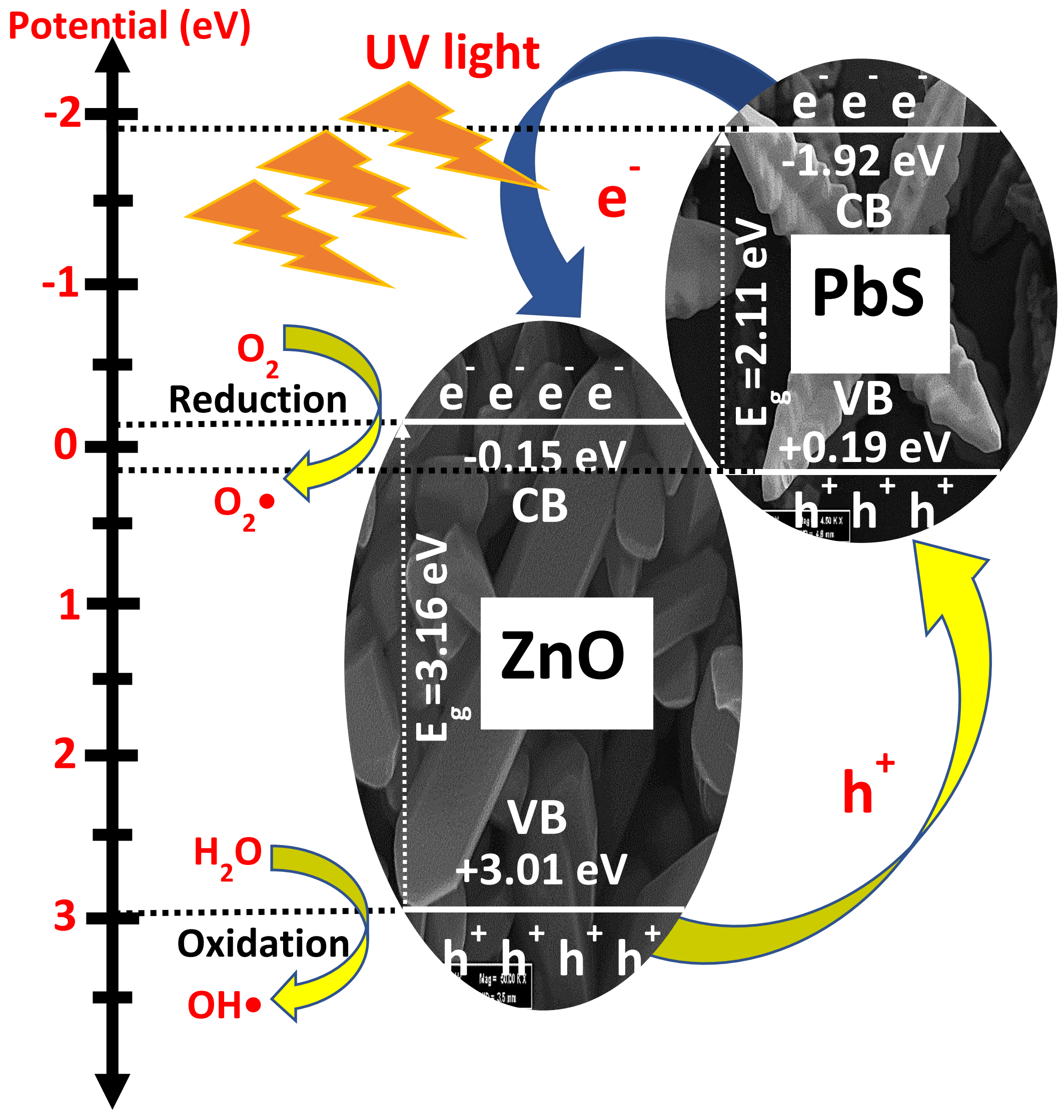

Figure S7. A proposed photo-catalytic mechanism of dye degradation on the surface of the PbS@ZnO heterojunction semiconductor photocatalyst.

Table S2. Comparison of the Photocatalytic Removal Rates of the Heterojunction Photocatalysts in This Study with the Results of Previous Studies Conducted Under Similar Experimental Conditions in the Relevant Field.

Catalyst	Irradiation time (min)	Dosage	Removal efficiency (%)	Reference
P-25 Degussa TiO_2	360	1200 mg/L	100	[87]
ZnO NPs	120	10-20 mg/L	90	[88]
ZnO:Eu NPs	150	1000 mg/L	90	[89]
$MnTiO_3$ NPs	240	100 mg/L	70	[90]
$MnTiO_3$-TiO_2 NPs	240	100 mg/L	75	
MWCNT/TiO_2 NPs	100	500 mg/L	76	[91]
ZnO/$NiFe_2O_4$ NPs	70	400 mg/L	~ 100	[92]
Fe/ZnO/SiO_2 NPs	30	75 mg/L	100	[93]
Fe_2O_3/TiO_2 NPs	60	10 mg/L	~ 95	[94]
Cu doped ZnS NPs	180	1000 mg/L	100	[95]
**ZnS@ZnO NPs**	480	666 mg/L	95.3	[**This study**]
**CdS@ZnO NPs**			90.5	
**PbS@ZnO NPs**			89.4	
